# Mining Symbionts of a Spider‐Transmitted Fungus Illuminates Uncharted Biosynthetic Pathways to Cytotoxic Benzolactones

**DOI:** 10.1002/anie.201916007

**Published:** 2020-03-18

**Authors:** Sarah P. Niehs, Benjamin Dose, Sophie Richter, Sacha J. Pidot, Hans‐Martin Dahse, Timothy P. Stinear, Christian Hertweck

**Affiliations:** ^1^ Department of Biomolecular Chemistry Leibniz Institute for Natural Product Chemistry and Infection Biology (HKI) Beutenbergstr. 11a 07745 Jena Germany; ^2^ Department of Microbiology and Immunology Doherty Institute 792 Elizabeth Street Melbourne 3000 Australia; ^3^ Department of Infection Biology HKI Germany; ^4^ Faculty of Biological Sciences Friedrich Schiller University Jena 07743 Jena Germany

**Keywords:** biosynthesis, drug discovery, genome mining, natural products, symbiosis

## Abstract

A spider‐transmitted fungus (*Rhizopus microsporus*) that was isolated from necrotic human tissue was found to harbor endofungal bacteria (*Burkholderia* sp.). Metabolic profiling of the symbionts revealed a complex of cytotoxic agents (necroximes). Their structures were characterized as oxime‐substituted benzolactone enamides with a peptidic side chain. The potently cytotoxic necroximes are also formed in symbiosis with the fungal host and could have contributed to the necrosis. Genome sequencing and computational analyses revealed a novel modular PKS/NRPS assembly line equipped with several non‐canonical domains. Based on gene‐deletion mutants, we propose a biosynthetic model for bacterial benzolactones. We identified specific traits that serve as genetic handles to find related salicylate macrolide pathways (lobatamide, oximidine, apicularen) in various other bacterial genera. Knowledge of the biosynthetic pathway enables biosynthetic engineering and genome‐mining approaches.

## Graphical abstract

**Spider sense**: The fungus *Rhizopus microsporus*, isolated from necrotic tissue resulting from a spider bite, was found to contain bacteria (*Burkholderia* sp.) that produce a complex of highly cytotoxic compounds (necroximes). Analysis of the PKS/NRPS hybrid assembly line and genome mining revealed the molecular basis of the biosynthesis of V‐ATPase‐inhibiting antitumor agents from diverse biological origins.

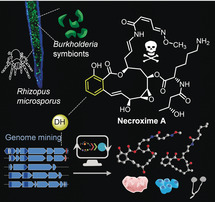

A brief encounter between a spider and an Australian woman in her garden in the late 1980s caused immense suffering to the latter. It started with a single prick in her little finger, which led to a mixed infection followed by necrosis that could only be stopped by multiple doses of amphotericin and below‐elbow amputation of the right hand. Cultures of samples taken from deep necrotic tissue grew a fungus that was later identified as the zygomycete fungus *Rhizopus microsporus* Tieghem var. *microsporus* CBS 308.87.^1^ Fungal strains of this species are well known as plant pathogens that cause, among others, rice seedling blight.^2^ Beyond plant diseases, there have been severe incidents of human infections, in particular in immunocompromised patients, some of which led to necroses in infected areas.^3^ Insects and spiders have repeatedly been reported as vectors that implant zygomycete spores into subcutaneous tissue.^3^

We have discovered that certain *R. microsporus* strains harbor bacteria that live within the fungal hyphae.^4^ These bacterial endosymbionts have been found to be responsible for the production of the antimitotic rhizoxin complex, the causative agent of rice seedling blight, which had initially been attributed to the fungi.^5^ Furthermore, the endofungal bacteria control their hosts’ vegetative reproduction through spores, which facilitates the symbiosis and its dissemination across the globe.^6^ It is intriguing that the fungal strain that was transmitted by the spider bite contains endofungal bacteria too.^7^ These bacterial endosymbionts (*Burkholderia* sp. HKI‐0404, isolate B8; Figure 1 A), however, comprise a phylogenetically distinct clade of the *Rhizopus* symbionts and are only remotely related to the other seven studied isolates from other continents.^7^ Apart from their ability to produce the antimitotic rhizoxin complex,^8^ the full metabolic potential of the B8 endosymbionts has remained elusive. Herein, we report the discovery and full characterization of novel cytotoxic benzolactones and elucidate their biosynthetic pathways from the spider‐transmitted *Rhizopus* endosymbionts. Using genomics, we identify a biosynthetic signature of related biosynthetic gene clusters in diverse bacterial phyla, with implications for understanding the origins of benzolactones from diverse sources.

**Figure 1:**
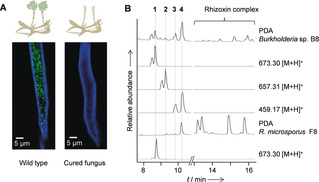
Bacterial endosymbionts of spider‐transmitted fungus and their metabolic profiles. A) *Burkholderia* sp. strain B8 and *Rhizopus microsporus* monitored by fluorescence microscopy: staining with Calcofluor White and Syto9 Green. B) HPLC profiles of *Burkholderia* sp. in pure culture (B8) and in symbiosis with the host (F8): extracted ion chromatograms [*M*+H]^+^ or PDA (200–600 nm).

To uncover natural products that could have contributed to the necrosis caused by the spider‐transmitted fungal infection, we compared the metabolic profiles of eight different isolates of bacterial symbionts. We found that the Australian isolate *Burkholderia* sp. B8 produces the antimitotic rhizoxin complex at lower titers than strains B1–B7, which are associated with hosts from geographically distant areas. Furthermore, HPLC analyses revealed a family of compounds that are unique to strain B8 (Figure 1 B). MS network analysis indicated the presence of several congeners, and their similar MS/MS fragmentation patterns pointed to the presence of stereoisomeric pairs of congeners. A series of optimizations, the use of absorber resin, adjusting preparative HPLC conditions, and highly reduced acidic conditions during the workup, eventually enabled the isolation of the main components (**1**–**4**) from the extract of an upscaled symbiont culture (7 L).

The pure compounds were subjected to a panel of cytotoxicity assays probing the antiproliferative effects against mammalian cell lines (HUVEC, K‐562, THP‐1, HEK‐293) and cytotoxicity against HeLa cells. We found that **1** is the most potent against HUVEC cells (GI_50_ 0.60 μm), whereas **4** most effectively inhibits the growth of THP‐1 cells (GI_50_ 0.44 μm). All compounds are highly cytotoxic against HeLa cells (CC_50_ **1**: 1.93 μm, **3**: 0.87 μm, and **4**: 1.09 μm). In addition, **1** selectively inhibits the yeast‐like fungus *Sporobolomyces salmonicolor*, whereas other tested fungal and bacterial strains are not affected. The marked cytotoxicity of the compounds suggests that they may have contributed to the formation of necrotic tissue following spider‐transmitted *Rhizopus* infection. To scrutinize this scenario, we analyzed the metabolic profile of the holobiont and found that these bacteria‐derived cytotoxins are also produced in symbiosis with the fungal host (Figure 1 B).

The structures of compounds **1**–**4** were fully elucidated by 1D and 2D NMR experiments and chemical derivatization (Figure 2 A). According to chemical shifts and 2D NMR correlations, **1** and **2** have a benzolactone core structure with an oxime side chain that corresponds to the planar structure of CJ‐12,950 (**5**; Figure 2 B), a natural product isolated from the zygote fungus *Mortierella verticillata* NRRL6337.^9^ However, NMR and MS data indicated that **1** and **2** have additional side chains attached to C‐13, according to long‐range couplings from H‐13 to C‐24 across the ester bonds. In compound **1**, the side chain consists of a lysine residue connected to a 3‐hydroxybutyric acid moiety. HRMS data, MS fragmentation, and NMR correlations revealed that **2** is a deoxy derivative of **1** in which the 3‐hydroxybutyryl is replaced by a butyryl residue (Figure 2 A). The smaller congeners, compounds **3** and **4**, have planar structures that match those reported for the 20*Z*‐ and 20*E*‐isomers CJ‐12,950 (**5**) and CJ‐13,357 (**6**) from *M. verticillata*.^9^

**Figure 2:**
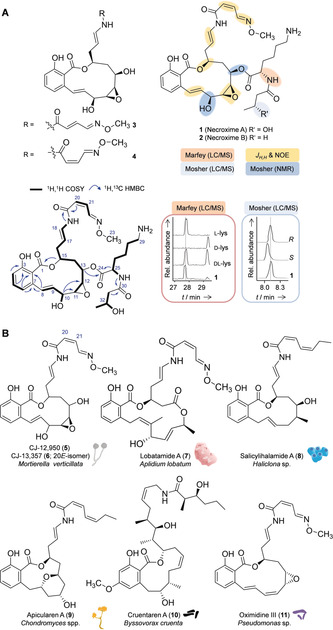
Structures of symbiont‐derived benzolactones and related natural products. A) Structure elucidation of necroximes A–D (**1**–**4**), selected 2D NMR data, and experiments to confirm absolute configurations. B) Benzolactone structures from diverse producer strains.

Since only the two‐dimensional structures of **5** and **6** had been reported,^9^ we set out to elucidate the configurations of all stereocenters of the symbiont metabolites. We identified the absolute configurations of the OH groups at C‐10 (*S*) and C‐13 (*R*) by Mosher ester analysis. NOE couplings indicated that the lactone hydroxy group at C‐15 is *S*‐configured and corroborated the architecture of the *cis*‐epoxide (*J*_11‐12_=4.0 Hz), with additional NOE signals of H‐12 and H‐11 confirming a similar orientation to H‐10, H‐13, and H‐15 on the macrocyclic ring. Using Marfey's reagent, we determined the configuration of lysine (l) in the side chain of **1**, and Mosher ester analysis in combination with LC revealed *R* configuration for the hydroxybutyrate moiety (Figure 2 A). Due to their origin and prominent structural motif, we named the new compounds necroximes A–D (**1**–**4**).

The necroximes are new additions to the family of bacterial salicylate macrolides, also referred to as benzolactone enamides, which act as selective inhibitors of mammalian vacuolar‐type (H^+^)‐ATPases (V‐ATPases) and thus effectively inhibit tumor cell growth.^10^ The origins of benzolactone enamides are surprisingly diverse: in addition to the fungi‐derived compounds **5** and **6**, important examples of these natural products are lobatamide (**7**) from a tunicate,^11^ salicylihalamide (**8**) from a sponge,^12^ apicularen (**9**) and cruentaren A (**10**) from myxobacteria,^13^ and oximidines (**11**) from pseudomonads^14^ (Figure 2 B). Considering the clinical importance of benzolactone enamides and the range of known structures, it is remarkable that to date the biosynthesis of the entire class of bacterial benzolactones has remained elusive.

To gain insight into the molecular basis of necroxime biosynthesis, we sequenced the genome of *Burkholderia* sp. B8 using the Illumina NextSeq platform. In the genome of strain B8, we identified a 66 kb gene locus (see the Supporting Information; Gene accession number MN734804) that appeared to be the best candidate for the necroxime (*nec*) biosynthetic gene cluster (Figure 3 A). The *nec* gene locus comprises genes (*necA–J*) for a multimodular *trans*‐AT PKS/NRPS hybrid assembly line that is largely colinear with the backbone of the necroximes. An encoded cytochrome P450 monooxygenase (NecI) is likely involved in the final oxygenations. Furthermore, a phylogenetic analysis of the ketosynthase (KS) domains of the encoded PKS modules^15^ revealed KS substrate specificities that match with predicted biosynthetic intermediates. Likewise, computational analyses of the NRPS adenylation domain specificities^16^ corroborated the correct building blocks. The absolute configurations of the ketoreductase (KR)‐generated hydroxy groups were confirmed by bioinformatics (Supplementary Information).

**Figure 3:**
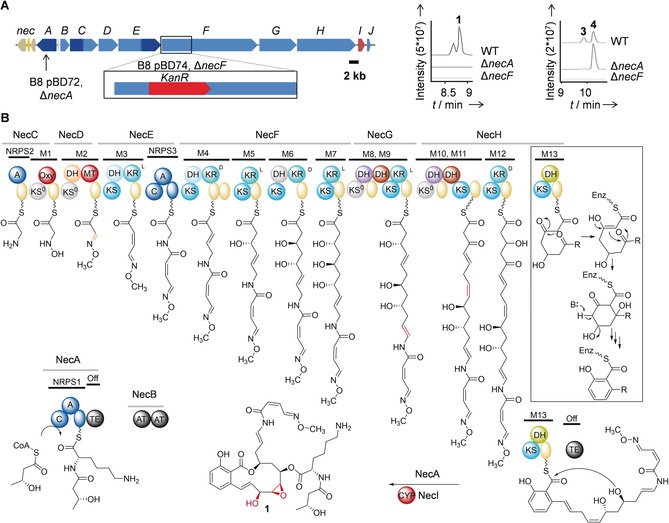
Molecular basis of necroxime biosynthesis. A) Organization of the *nec* biosynthetic gene cluster, strategy for gene deletion, and metabolic profiles of mutants. KanR=kanamycin resistance cassette. Dark blue: NRPS genes, light blue: PKS genes, orange: a regulatory gene, red: a cytochrome P450 monooxygenase gene, gray/light brown: hypothetical/additional genes. B) Model for necroxime biosynthesis on a PKS/NRPS assembly line. KS=ketosynthase (cyan for canonical; gray for non‐elongating); KR=ketoreductase; DH=dehydratase (light blue for canonical; dark red for double‐bond shift; violet and orange for non‐canonical); Oxy=oxygenase; MT=methyltransferase; TE=thioesterase; CYP=cytochrome P450 monooxygenase; ACP (yellow sphere)=acyl carrier protein; C=condensation; A=adenylation; PCP (blue sphere)=peptidyl carrier protein; AT=acyl transferase; gray spheres=possibly inactive domains.

To unequivocally assign the *nec* gene cluster to necroxime production, we created targeted gene‐deletion mutants of strain B8 (Δ*necA* and Δ*necF*) by using knockout plasmids based on the enhanced double‐selection plasmid pGL42a^17^ (Figure 3 A), and verified them by PCR. Since the metabolic profile of Δ*necF* was devoid of necroximes, the identity of the first gene cluster responsible for the production of a benzolactone enamide was proven. Moreover, while Δ*necA* was unable to produce necroximes A (**1**) and B (**2**), necroximes C (**3**) and D (**4**) were still observed in extracts of this mutant (Figure 3 A), thus indicating that NecA is responsible for the formation and attachment of the peptide side chain in **1** and **2**.

The deduced assembly line features a number of unusual PKS domains and module architectures that account for the structural signatures of the necroximes (Figure 3 B). The first three modules are obviously in charge of installing the rare oxime side chain by means of specialized oxygenase (OXY), dehydratase (DH), and methyltransferase (MT) domains that modify the side chain of the glycine primer. In addition to the tentative oxime‐forming DH, the PKS harbors a number of non‐canonical DH domains and module architectures, such as two DH domains in one module (M8–11). To learn more about the functions of these DH domains, we performed a multiple sequence alignment and inferred a phylogeny (Figure 4 A and the Supporting Information). The DH domains cluster into four different groups in which some of the sequences deviate from the typical catalytic dyad.^18^ DH domains from modules 9 and 11 fall into a clade with so‐called double‐bond shift domains^19^ and may thus be involved in two double‐bond shifts during necroxime biosynthesis. The most unusual DH domain, however, is located in the terminal PKS module, which is implicated in the formation of the salicylate moiety (Figure 3 B).

**Figure 4:**
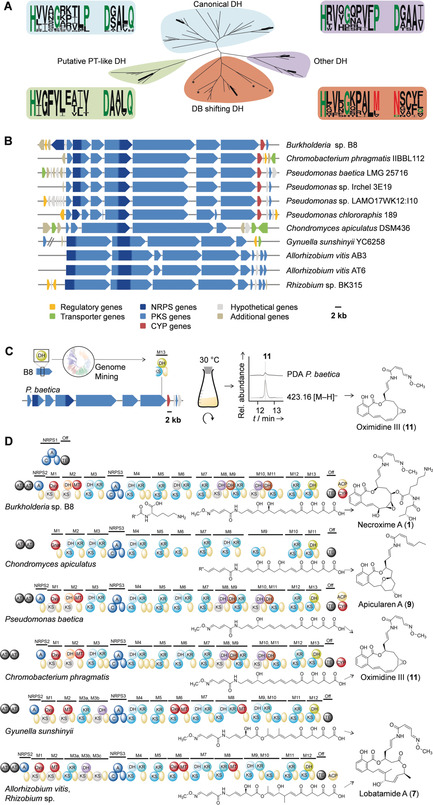
Genome mining for benzolactone assembly lines. A) Phylogenetic analysis of DH domains and their conserved motifs (Weblogo) and comparison of canonical (HXXXGXXXXP and DXXXQ) and non‐canonical bacterial DH domains. DB=double bond. Asterisks indicate DB‐shift domains from other assembly lines. DH domains from *nec* PKS are shown in bold. DH2 (orange) was omitted from the analyses. B) Organizations of tentative benzolactone biosynthesis gene clusters identified by genome mining using PT‐like DH sequence. C) Genomics‐guided identification of oximidine III in *P. baetica*. D) Deduced PKS/NRPS assembly lines and predicted metabolite backbones that correspond to known benzolactones. Color code as in Figure 3.

To date, salicylate macrolactone biosynthesis has been exclusively studied in fungi. In the zearalenone, hypocemycin, and radicicol biosynthetic pathways,^20^ the salicylate ring is formed through the action of a so‐called product template (PT) domain.^21^ No equivalents to these PT domains have been reported for bacterial modular PKSs. Notably, DH and PT domains evolved from a common ancestor since the two families have conserved catalytic dyads and tertiary structures.21b Therefore, the DH in the terminal module (M13) could, in principle, meet the requirements to catalyze salicylate formation in a manner analogous to the fungal pathways.

Since the motifs of this DH domain are markedly different from the canonical ones (Figure 4 A), we employed the amino acid sequence of the terminal DH as a bioinformatic handle to screen bacterial genomes for related biosynthetic machineries (Figure 4 B). By means of this genome‐mining approach, we identified ten related PKS/NRPS gene clusters that tentatively code for salicylate macrolactones. In the genomes of one *Chromobacterium* sp. and four *Pseudomonas* spp., we identified gene clusters that could code for the biosynthesis of oximidines (**11**).^14^ In contrast to the necroximes, the oximidines lack an N‐acylated lysine side chain, which is reflected by the missing NRPS1 gene. As a proof of concept, we investigated culture extracts of the fish pathogen *Pseudomonas baetica* and found that these bacteria indeed produce oximidine III (Figure 4 C). Furthermore, we detected an orthologous gene cluster in the genome of the apicularen producer *Chondromyces apiculatus* (Figure 4 D). Notably, apicularen A (**9**) is a benzolactone that lacks the oxime moiety. In agreement with the prediction, the deduced assembly line lacks the designated domains for oxime formation and is colinear with the apicularen backbone. Next, four related gene clusters were identified in the genomes of two *Allorhizobium* spp., a *Rhizobium* sp., and the rhizosphere bacterium *Gynuella sunshinyii*, which has been identified as a promising source of natural products.^22^ The deduced PKS/NRPS assembly lines, which harbor additional oxygenase domains that may insert oxygens into the polyketide backbone, code for the biosynthesis of lobatamide or derivatives thereof (**7**; Figure 4 D).^23^ Based on the high conservation of the biosynthetic machineries exclusively among bacteria, we suggest that endosymbionts are the true producers of lobatamide in the tunicate *Aplidium lobatum*.^11^ Likewise, we propose that salicylihalamide (**8**) from the demosponge *Haliclona* sp.12a and the CJ compounds (**5** and **6**) from the fungus *M. verticillata* 9 are of bacterial origin.

In conclusion, from the endobacteria of a spider‐transmitted fungus, we isolated and characterized a complex of cytotoxic agents, two of which represent the largest known bacterial benzolactone oximes, a class of natural products with high potential for antitumor therapies. Despite their pharmacological relevance, the biosynthetic machineries responsible for macrolactone production have remained enigmatic. Herein, we unravel the molecular basis for bacterial benzolactone formation and provide the first model for their biosynthesis, which involves unusual oxime formation and aromatization steps. The knowledge of the biosynthetic pathway sets the stage for future bioengineering and genome‐mining approaches. As a proof of concept, we showed that an unprecedented PT‐like DH domain can be used to discover cryptic gene clusters in diverse bacterial genera that might code for various known V‐ATPase inhibitors. This finding challenges the biogenetic origin of several natural products previously isolate from sponges, tunicates, and fungi, and suggests that they may actually be biosynthesized by bacterial endosymbionts. Thus, beyond setting an unusual example of hypothesis‐based drug discovery in the context of human disease, our work has implications for biosynthetic engineering, genome mining, and ecology.

## Conflict of interest

The authors declare no conflict of interest.

## Supporting information

As a service to our authors and readers, this journal provides supporting information supplied by the authors. Such materials are peer reviewed and may be re‐organized for online delivery, but are not copy‐edited or typeset. Technical support issues arising from supporting information (other than missing files) should be addressed to the authors.

Supplementary
