# Supplementary material for: Mining Symbionts of a Spider‐Transmitted Fungus Illuminates Uncharted Biosynthetic Pathways to Cytotoxic Benzolactones
Source: Angew Chem Int Ed Engl. 2020 Mar 18;59(20):7766–71. doi: 10.1002/anie.201916007 (PMC7318616; doi:10.1002/anie.201916007)
Supplement: Supplementary file 1 — Supplementary [file ANIE-59-7766-s001.pdf]

Angewandte Chemie
Eine Zeitschrift der Gesellschaft Deutscher Chemiker

Supporting Information

Mining Symbionts of a Spider-Transmitted Fungus Illuminates Uncharted Biosynthetic Pathways to Cytotoxic Benzolactones

*Sarah P. Niehs, Benjamin Dose, Sophie Richter, Sacha J. Pidot, Hans-Martin Dahse, Timothy P. Stinear, and Christian Hertweck**

anie_201916007_sm_miscellaneous_information.pdf

Table of contents

Experimental procedures

Table of contents 1
Experimental procedures 1
Supplemental tables 2
Bacterial and fungal strains 3
Identification and extraction of secondary metabolites 4
Necroxime isolation and purification 4
Identification and annotation of necroxime biosynthesis gene cluster 5
Genetic manipulation of *Burkholderia* sp. HKI-0404 (strain B8) 7
Phylogenetic analyses 12
General analytical methods 17
Determination of the absolute configurations 18
Determination of bioactivity 20
NMR Tables 22
MS/MS spectra 28
NMR spectra 31
References 54
Author contributions 54

Supplemental figures

Figure S1. PCR-based verification of gene inactivations 8
Figure S2. Extracted catalytic dyads from DH domains 12
Figure S3. Phylogenetic analysis of DH domains 13
Figure S4. Phylogenetic analysis of substrate-specific ketosynthase domains. 14
Figure S5. Phylogeny of fungal DH and PT (product template) domains 16
Figure S6. Agar diffusion assay of necroxime A against *S. salmonicolor*. 21
Figure S7. Structure of necroxime A with NMR assignments. 23
Figure S8. MS/MS fragmentation pattern of *m/z* 673.3071 $[M+H]^+$, necroxime A (**1**). 28
Figure S9. MS/MS fragmentation pattern of *m/z* 423.1560 $[M–H]^–$ 28
Figure S10. MS/MS fragmentation pattern of *m/z* 457.1615 $[M–H]^–$ 29
Figure S11. MS/MS fragmentation pattern of m/z 657.3132 $[M+H]^+$ 29
Figure S12. MS/MS fragmentation pattern of *m/z* 459.1833 $[M+H]^+$ 30
Figure S13. MS/MS fragmentation pattern of *m/z* 459.1758 $[M+H]^+$ 30
Figure S14. ^{1}H NMR spectrum of necroxime A. 31
Figure S15. ^{13}C NMR spectrum of necroxime A. 31
Figure S16. ^{1}H-^{1}H COSY NMR spectrum of necroxime A. 32
Figure S17. DEPT-135 NMR spectrum of necroxime A. 33
Figure S18. ^{1}H-^{13}C HMBC NMR spectrum of necroxime A. 34
Figure S19. ^{1}H-^{13}C HSQC NMR spectrum of necroxime A. 35
Figure S20. ^{1}H-^{1}H NOESY NMR spectrum of necroxime A. 36
Figure S21. ^{1}H-^{1}H TOCSY NMR spectrum of necroxime A. 37
Figure S22. ^{1}H NMR spectrum of necroxime B. 38
Figure S23. ^{13}C NMR spectrum of necroxime B. 38
Figure S24. DEPT-135 NMR spectrum of necroxime B. 39
Figure S25. ^{1}H,^{1}H COSY NMR spectrum of necroxime B. 40

Figure S26. $^{1}H,^{1}H$ NOESY NMR spectrum of necroxime B. 41
Figure S27. $^{1}H,^{1}H$ TOCSY NMR spectrum of necroxime B. 42
Figure S28. $^{1}H,^{13}C$ HSQC NMR spectrum of necroxime B. 43
Figure S29. $^{1}H,^{13}C$ HMBC NMR spectrum of necroxime B. 44
Figure S30. ^{1}H NMR spectrum of necroxime C. 45
Figure S31. ^{13}C NMR spectrum of necroxime C. 45
Figure S32. ^{1}H-^{1}H COSY NMR spectrum of necroxime C. 46
Figure S33. DEPT-135 NMR spectrum of necroxime C. 47
Figure S34. ^{1}H-^{13}C HMBC NMR spectrum of necroxime C. 48
Figure S35. ^{1}H-^{13}C HSQC NMR spectrum of necroxime C. 49
Figure S36. ^{1}H NMR spectrum of necroxime D. 50
Figure S37. ^{13}C NMR spectrum of necroxime D. 50
Figure S38. $^{1}H,^{1}H$ COSY NMR spectrum of necroxime D. 51
Figure S39. $^{1}H,^{13}C$ HMBC NMR spectrum of necroxime D. 52
Figure S40. $^{1}H,^{13}C$ HSQC NMR spectrum of necroxime D. 53

Supplemental tables

Table S1. Overview of bacterial and fungal strains. 3
Table S2. Residues in binding pocket of adenylation domains. 5
Table S3. Origin of putative benzolactone producers and genome accession numbers. 6
Table S4. Primer used for this study. 9
Table S5. Gene-deletion mutants generated for this study. 9
Table S6. Deduced gene functions. 10
Table S7. Conserved amino acid residues coding for ketoreductase amino acid specificity... 15
Table S8. Results of Mosher ester analysis. 19
Table S9. Results from agar diffusion assays. 20
Table S10. Cytotoxic and antiproliferative properties of the necroximes 21
Table S11. NMR shifts of necroxime A (**1**). Measured in DMSO-d_6. 22
Table S12. NMR shifts of necroxime B (**2**). Measured in DMSO-d_6. 24
Table S13. NMR shifts of necroxime C (**3**). Measured in DMSO-d_6. 26
Table S14. NMR shifts of necroxime D (**4**). Measured in DMSO-d_6. 27

Experimental procedures

Bacterial and fungal strains

Strains of this study are listed in Table S1.

Burkholderia sp. HKI0404 (strain B8) was isolated from *Rhizopus microsporus* Tieghem var. *microsporus* CBS 308.87 according to the following protocol: the fungus was inoculated in MGY+M9 medium[1] and grown at 30 °C and 110 rpm until the supernatant was turbid. The culture was centrifuged at 8,800 × *g*, at room temperature for 10 min. Approximately 10 µL were spread on NAG agar (Standard nutrient agar, Merck, +1% glycerol). The plates were inoculated at 30 °C and checked for bacterial colonies after 3–4 days. Fungal strains were kept on potato dextrose agar (BD, Bacto) for short-time storage at room temperature. For long-time storage, the fungal spores or the bacterial strains were stored in 25% glycerol at –20 °C.

Table S1. Overview of bacterial and fungal strains.

Strain	No.	Original site of isolation
Pseudomonas baetica	DSM 26532 (CECT 7720, LMG 25716)	Liver of a diseased wedge sole, South-west Spain
Burkholderia sp.	HKI0404, strain B8	*R. microsporus* CBS 308.87
Rhizopus microsporus Tieghem var. *microsporus*	CBS 308.87 (NRRL 28628)	Necrotic human tissue resulting from spider bite, Australia
Escherichia coli	TOP10	-

Fluorescence microscopy: Visualization of endosymbionts in the fungal hyphae was conducted using Calcofluor White Stain (Sigma) in combination with SYTO 9 Green Fluorescent Nucleic Acid Stain (Invitrogen). A small piece of fungal hyphae was stained in the dark for 5 min, washed with 0.85% NaCl solution, and examined for the presence of endosymbionts at a Zeiss CLSM 710 confocal laser-scanning microscope (at wavelength recommended by the manufacturer).

Preparation of aposymbiotic strain: A small piece of fungal hyphae was used to inoculate on potato dextrose agar (BD, Bacto) supplemented with either ciprofloxacin (40 µg mL^{-1}) or kanamycin (50 µg mL^{-1}) and continuously incubated. The absence of bacterial endosymbionts

in the fungus was verified by confocal laser-scanning microscope and metabolic profiling by LC/MS.

Identification and extraction of secondary metabolites

Pseudomonas baetica DSM 26532 was cultivated in 40 mL M9 medium (per liter: 10 g glycerol, 7 g K_2HPO_4, 2 g KH_2PO_4, 0.59 g sodium citrate, 1 g $(NH_4)_2SO_4$, 0.1 g $MgSO_4$) with a start OD_{600} 0.05 at 30 °C, 110 rpm for 4 days. The cultures were extracted with 1:1 volume of ethyl acetate overnight. Subsequently, the organic phase was dried with Na_2SO_4, concentrated under reduced pressure and the residue was dissolved in methanol.

Burkholderia sp. strain B8 (HKI-0404) was cultivated in 50 mL MGY+M9 medium[1] (addition of 1% XAD-2 resin) at 30 °C and 110 rpm for 5 days. The resin was separated from the culture broth, washed with water and extracted in methanol for 1 hour. Following, the organic phase was concentrated under reduced pressure and the residue was dissolved in methanol.

The extracts were measured with HRESI/MS.
Measured *m/z* 425.1705 $[M+H]^+$, calculated 425.1707, $C_{23}H_{25}N_2O_6$ (oximidine II/III)
Measured *m/z* 459.1833 and 459.1758 $[M+H]^+$,
calculated 459.1762, $C_{23}H_{27}N_2O_8$ (necroxime C and D)
Measured *m/z* 673.3088 $[M+H]^+$, calculated 673.3079, $C_{33}H_{45}N_4O_{11}$ (necroxime A)
Measured *m/z* 657.3129 $[M+H]^+$, calculated 657.3130, $C_{33}H_{45}N_4O_{10}$ (necroxime B)

Necroxime isolation and purification

Burkholderia sp. strain B8 culture up-scaled to 50 mL MGY+M9 medium[1]. 400 mL MGY+M9 medium (medium with addition of 1% XAD-2 resin) per 1 L flask was inoculated with bacteria and incubated until OD_{600} 0.05 at 30 °C and 110 rpm for 6–7 days (overall 7 L).

The absorber resin was extracted with 100% methanol overnight. The extract was concentrated under reduced pressure and the residue dissolved in a small volume of methanol. Then, a Sephadex LH-20 column was applied with 100% methanol as eluent. Final purification was conducted *via* preparative HPLC: Luna column (10 µm C18(2) 100 Å 250 × 21.2 mm, phenomenex), flow rate: 15 mL min^{-1}, solvents: water supplemented with 0.01% trifluoroacetic acid and methanol, gradient: 0–5 min 15% methanol, 5–40 min 15% to 100% methanol.

Identification and annotation of necroxime biosynthesis gene cluster

Burkholderia sp. HKI0404 (strain B8) was grown in MGY+M9 medium at 30 °C and 110 rpm until the late exponential growth phase was reached. Genomic DNA of *Burkholderia* sp. HKI0404 was extracted with the MasterPure DNA Purification Kit (Epicentre) according to manufacturer's recommendations. Strain B8 was sequenced on the Illumina NextSeq platform using the Nextera XT DNA preparation kit (Illumina) with 1 × 150 bp paired end chemistry. A sequencing depth of >50-fold was targeted for each sample. Illumina sequencing data were assembled with SPAdes (v 3.10.1)[2] and annotated with Prokka v 1.12.[3]

AntiSMASH v5[4] and PKS/NRPS analysis[5] were used for annotation of the benzolactone encoding biosynthetic gene cluster (Table S2).

Table S2. Residues in binding pocket of adenylation domains of benzolactone-producing assembly lines, and prediction of putative incorporated amino acids.

Organism	Residues in binding pocket of NRPS2	Prediction	Residues in binding pocket of NRPS3	Prediction
Burkholderia sp. HKI-0404	DILXICMI	100% Gly	DILQVSLI	100% Gly
Chondromyces apiculatus DSM436	-	-	DIMXFGLI	80% Gly
Chromobacterium phragmitis IIBBL112	DILQ-CMI	No hit	DILQLGLI	100% Gly
Pseudomonas baetica	DILXICMI	100% Gly	DILQXXLI	No hit
Pseudomonas chlororaphis isolate 189	DILXICMI	100% Gly	DILQVSLI	100% Gly
Pseudomonas sp. Irchel 3E19	DILXICMI	100% Gly	DILXICMI	100% Gly
Pseudomonas sp. LAMO17WK12:I10	DILXFGMI	62% Gly	DILQVSLI	100% Gly
Gynuella sunshinyii YC6258	DILQLGMI	100% Gly	DILQLGLI	100% Gly
Allorhizobium vitis AB3	DILQLGMI	100% Gly	DILQLGLI	100% Gly
Allorhizobium vitis AT6	DILQLGMI	100% Gly	DILQLGLI	100% Gly
Rhizobium sp. BK315	DILQLGMI	100% Gly	DILQLGLI	100% Gly

Table S3. Origin of putative benzolactone producers and genome accession numbers.

Organism	Origin	Accession number
Burkholderia sp. HKI-0404	Necrotic human tissue resulting from spider bite (Australia)	MN734804
Chondromyces apiculatus DSM436	Decayed wood, Minneapolis (USA)	GCF_000601485
Chromobacterium phragmitis IIBBL112	Tidal marsh of the Potomac river, Maryland (USA)	NZ_CP029495.1
Pseudomonas baetica	Liver of a diseased wedge sole (Spain)	NZ_PHHE00000000.1
Pseudomonas chlororaphis isolate 189	Soil near Golden Prairie (Canada)	CP014867.1
Pseudomonas sp. Irchel 3E19	Pond Irchel campus (Switzerland)	NZ_FYDT00000000.1
Pseudomonas sp. LAMO17WK12:I10	Unknown	OBDX01000097.1
Gynuella sunshinyii YC6258	Rhizosphere of the halophyte *Carex scabrifolia* Steud., tidal flat area (Korea)	CP007142.1
Allorhizobium vitis AB3	*Vitis vinifera*, crown gall tumor (Hungary)	NZ_MAVS01000011.1
Allorhizobium vitis AT6	*Vitis vinifera*, crown gall tumor (Hungary)	NZ_MBEX01000040.1
Rhizobium sp. BK315	Unknown	SLZI01000013.1

Genetic manipulation of *Burkholderia* sp. HKI-0404 (strain B8)

To establish a link between necroxime and the corresponding gene cluster, gene inactivation experiments were performed. Targeted gene inactivation was achieved by means of homologous recombination of the target genomic DNA with plasmids.

Cloning of the gene inactivation plasmids: Gene inactivation plasmids were constructed using a restriction ligation approach. The following conditions were used for all PCR reactions if not stated otherwise: OneTaq® 2X Master Mix with Standard Buffer (New England Biolabs) and 35 cycles of 95 °C for 30 s, 60 °C for 30 s kb^{-1}, and a final extension time at 72 °C for 300 s. Templates and primers are further specified down below. All enzymes have been purchased from New England Biolabs if not stated otherwise. Reactions have been conducted according to manufacturer's recommendations. All constructed plasmids were subjected to enzymatic digestions as well as sequencing to confirm the sequences.

Construction of pBD72:
The PCR products using the primer pair BD342 and BD343 and genomic DNA isolated from *Burkholderia* sp. HKI-0404 (strain B8) were blunt-end ligated into the linear pJet1.2 vector according to the manufacturer's recommendations. A kanamycin resistance cassette amplified from pGEM-Kan with the primers BD259 and BD260, as well as the vector were digested with *Kpn*I and subsequently mixed in the ligation buffer. The resulting plasmid was digested with *Xba*I and *Xho*I. The DNA fragment of 4,103 bp was purified from an agarose gel. The *Xba*I and *Pst*I digested double selection plasmid pGL42a T251A[6] was ligated with the 4,103 bp DNA fragment and yielded pBD72.

Construction of pBD74:
The vector (pGL42a T251A) was digested with *Xba*I and *Kpn*I and purified by gel electrophoresis. The PCR products amplified using the primers BD361 and BD362 from genomic DNA isolated from *Burkholderia* sp. HKI-0404 were digested with *Xba*I and *Kpn*I and ligated into the vector. The resulting plasmid was digested with *Bam*HI. A PCR-amplified kanamycin resistance cassette using BD357, BD358 and the template pGEM-Kan was also digested with *Bam*HI.[7] The ligation reaction of the digested fragments yielded pBD74.

Transformation of *Burkholderia* sp. HKI0404 by electroporation: *Burkholderia* sp. HKI0404 (strain B8) cells were transformed with plasmids pBD72 and pBD74 to inactivate the NRPS gene (*necA*) and one PKS gene (*necF*), respectively. *Burkholderia* cell cultures were inoculated with overnight pre-cultures (OD_{600} ~0.1) and grown until OD_{600} 0.6. The cells were centrifuged and washed three times with 1 volume of 300 mM sucrose solution. Afterwards, the cells were suspended in sucrose solution. 100 µL of the cell solution was transferred into an electroporation cuvette and mixed with 2 µL of a 100 ng $µL^{-1}$ plasmid in dH_2O solution. Electroporation at 2,500 V was applied before the cells were suspended in 500 µL MGY+M9 media. After 3–4 h incubation at 30 °C and 140 rpm the transformed cells were plated on NAG

plates with 50 µg mL^{-1} kanamycin and incubated at 30 °C until colonies appeared. Colonies were transferred double selection plates (MCGA plates with 50 µg mL^{-1} kanamycin)[1] and cultivated until mutants were detected by colony PCRs.

Gene knockout in *Burkholderia* sp. HKI0404: Homologous recombination of the knockout plasmids (pBD72 and pBD74) with the corresponding regions in the genome yielded the respective gene inactivation mutants B8 pBD72 and B8 pBD74. To detect mutations, colony PCRs were performed. Bacterial colony material was added to the primers pairs BD344 and BD345 respectively or BD363 and BD364 and mixed into the PCR reaction master mix (Table S4). The amplification of fragments of the appropriate size for the mutants B8 pBD72 (4,981 bp for the mutant and 3,813 bp for wild type) and B8 pBD74 (3,911 bp for the mutant and 2,743 bp for wild type) implied that the resistance gene had inserted at the appropriate site in the genome (Figures S1A and B). Mutant strains were extracted as described before to further confirm successful mutation/gene inactivation.

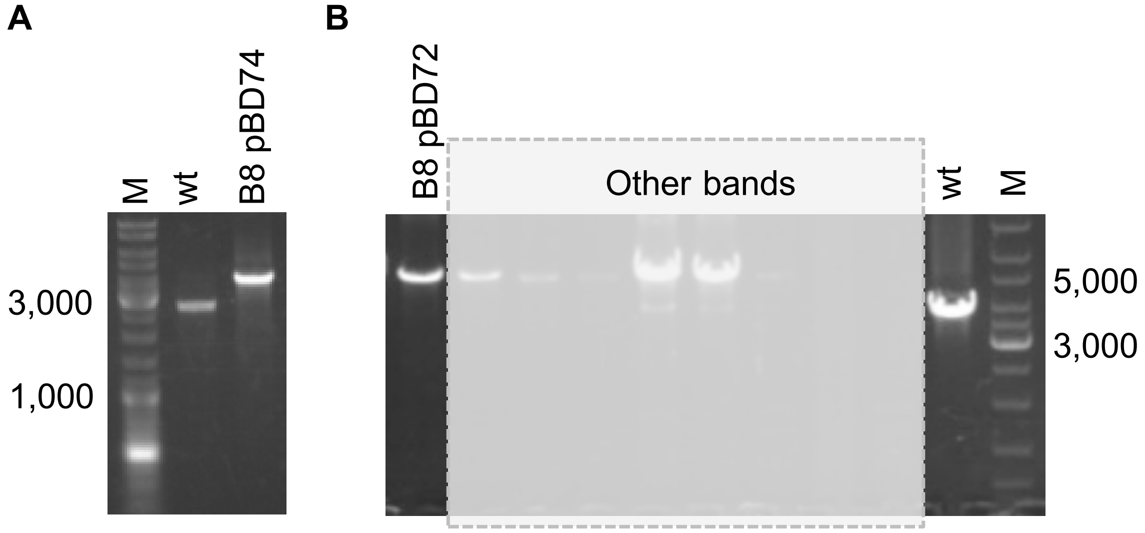

Figure S1. PCR-based verification of gene inactivations of (A) the thioesterase gene (*necA*) in B8 pBD72 and (B) the PKS gene (*necF*) in B8 pBD74.

Table S4. Primer used for this study.

Name	Sequence 5’→3’	Amplification of …
BD342	ttgagcgtatcgcgacatag	Homologous arms for NRPS gene knockout (pBD72)
BD343	ttgagcgtcgagattgtttg	
BD344	gcaagataagcggcagactc	Colony PCR to identify the genotypes
BD345	taagttcaccacgctgatgc	
BD259	atggtcggtaccgtaagcttaggctgctgcca	Amplification of kanamycin resistance cassette for pBD72
BD260	cgatcaggtacctcagaagaactcgtcaagaa	
BD361	aattcctgcagtacgacacacaatggcaagg	Homologous arms for PKS gene knockout (pBD74)
BD362	ggtggcggccgctgcaaaagtttgtccagacg	
BD363	gaacgcatagccaggtaacg	Colony PCR to identify the genotypes
BD364	gttatttgaaccgccctgag	
BD357	ctcgggatccgtaagcttaggctgctgcc	Amplification of kanamycin resistance cassette for pBD74
BD358	tcaaggatcctcagaagaactcgtcaag	

Table S5. Gene-deletion mutants generated for this study.

Name	Gene deletion	Effect
B8 pBD72	NRPS gene (*necA*)	Abolishment of necroxime A and B production, continued production of necroxime C and D
B8 pBD74	PKS gene (*necF*)	Abolishment of necroxime A–D production

Table S6. Deduced gene functions.

Locus tag B8_	bp	Deduced protein	Closest SwissProt entry	Acces-sion #	Organism	Identity/ Similar-ity [%]
orf+4	884	Lipid A biosynthesis lauroyl acyltransferase	Lipid A biosynthesis lauroyl acyltrans-ferase	P0ACV 1.1	*Escherichia coli* O157:H7	34/49
orf+3	878	Diaminopime-late epimerase	Diaminopime-late epimerase	Q2T26 9.1	*Burkholderia thailandensis* E264	77/85
orf+2	803	Hypothetical protein	-	-	-	-
orf+1	938	Tyrosine recombinase	Tyrosine Recomb-inase	B2U7W 2.1	*Ralstonia pickettii* 12J	58/72
necJ	770	4-Phospho-pantetheinyl transferase	4-Phospho-pantetheinyl transferase	P37695 .2	*Nostoc* sp. PCC 7120	36/52
necI	1400	Epi-isozizaen 5-monooxygenase	Cytochrome P450 monooxy-genase	Q9VYQ 7.1	*Drosophila melanogaster*	27/45
necH	11747	Polyketide syntase	Polyketide syntase	P40872 .4	*Bacillus subtilis* subsp. *subtilis* str. 168	31/47
necG	7379	Polyketide syntase	Polyketide syntase	P40806 .3	*Bacillus subtilis* subsp. *subtilis* str. 168	42/57
necF	18902	Polyketide syntase	Polyketide syntase	P40806 .3	*Bacillus subtilis* subsp. *subtilis* str. 168	31/48
necE	8486	Polyketide syntase	Polyketide syntase	O31782 .3	*Bacillus subtilis* subsp. *subtilis* str. 168	30/45
necD	3935	Polyketide syntase	Polyketide syntase	P40872 .4	*Bacillus subtilis* subsp. *subtilis* str. 168	28/44
necC	5580	Polyketide syntase	Polyketide syntase	P40806 .3	*Bacillus subtilis* subsp. *subtilis* str. 168	36/52

Table S6 continued. Deduced gene functions.

Locus tag B8_	bp	Deduced protein	Closest SwissProt entry	Acces-sion #	Organism	Identity/ Similar-ity [%]
necB	1896	Acyltrans-ferase	Polyketide biosynthesis malonyl CoA-acyl carrier protein transacylase	A7Z4X8.1	*Bacillus velezensis* FZB42	49/66
orf-1	333	Hypothetical protein	-	-	-	-
necA	4038	Modular NRPS	Modular NRPS	P45745.4	*Bacillus subtilis* subsp. *subtilis* str. 168	40/58
orf–2	249	Hypothetical protein	-	-	-	-
orf–3	1053	GTP binding protein	GTP binding protein	P24203.3	*Escherichia coli* K-12	41/58
orf–4	585	Transcription factor	Transcription factor RNA polymerase binding	B8H0C0.1	*Caulobacter vibrioides* NA1000	53/72
orf–5	537	ATP-dependent protease subunit	ATP-dependent protease subunit	Q146W9.1	*Paraburkholderia xenovorans* LB400	92/95
orf–6	1338	ATP-dependent protease subunit	ATP-dependent protease subunit	B2JJX7.1	*Paraburkholderia phymatum* STM815	89/94
orf–7	543	Regulatory protein photosynthesis apparatus	Regulatory protein photosynthesis apparatus	O82868.1	*Rhodovulum sulfidophilum*	47/62
orf–8	1269	Sensor histidine kinase	Sensor histidine kinase	Q3J6C1.1	*Rhodobacter sphaeroides* 2.4.1	29/43

Phylogenetic analyses

Multiple sequence alignment and phylogenetic analysis of dehydratase (DH) domains: The amino acid sequences of DH domains of (predicted) benzolactone-producing assembly lines were extracted and numerated according to the respective module (Figure S2). Double bond (DB) shifting DH as well as canonical DH domain sequences were taken from the biosynthesis gene cluster of rhizoxin (RhiE DH*, RhiF DH1, RhiD DH1, RhiD DH2),[8] corallopyronin (CorJ DH*),[9] bacillaene (BaeR DH*, BaeN DH3)[10] and difficidin (DifK DH*, DifF DH1, DifI DH1, DifI DH2).[11] Extensive gaps were manually deleted from the multiple sequence alignment. The sequences were subjected to a multiple sequence alignment using MAFFT 7[12] with default settings. A maximum likelihood phylogeny was constructed using IQ-tree.[13] Ultrafast bootstrapping (1,000 iterations) analysis was performed. The neighbour-joining tree construction method showed similar tree topology.

Sequence logos displaying the active center of DH domains: The active dyad sequence motif (the histidine in **H**XXXGXXXXP as well as the aspartate in **D**XXXQ) of the DH domains from the benzolactone-producing assembly lines were identified and submitted to WebLogo version 2.8.[14] Sequence logos for each clade of the DH phylogeny shown in Figure S3 were created. The sequences of the extracted motifs are listed down below in Figure S2.

Canonical DH domains

```
Pseubaet_DH6     1 HVVQGHHVLPXXXXXDGALQ
Chromo_DH6       1 HVIHGRRILPXXXXXDGALQ
B8_DH6           1 HLIHGRRILPXXXXXDGALQ
Chondapi_DH8     1 HVVQGRRTLPXXXXXDSLLQ
DhifI_DH2        1 HIVNGKPVLPXXXXXDAALQ
DifF_DH2         1 HIVNGKPVLPXXXXXDAALQ
RhiD_DH1         1 HVVGGRRIFPXXXXXDSALQ
RhiD_DH2         1 HQVDGCKTMPXXXXXDSAVQ
BaeN_DH3         1 HLIKGKPVMPXXXXXDSALQ
RhiF_DH1         1 HRVNGLKILPXXXXXDSALQ
DifF_DH1         1 HLVNEKYVLPXXXXXDGAVQ
Pseubaet_DH4     1 HLVSGAPTLLXXXXXEGALQ
Chromo_DH4       1 HIVSGAPTLLXXXXXDGALQ
D8_DH04          1 HVVSGAPTLLXXXXXDGALQ
Chondapi_DH4     1 HLVAGKKTLLXXXXXDGALQ
Chondapi_DH3     1 HVVAQRKVLPXXXXXDSALH
Psubaet_DH3      1 HVIAGRKTLPXXXXXDSAFQ
B8_DH3           1 HVIVERKTLPXXXXXDSAFQ
Chromo_DH3       1 HVVAGRKTLPXXXXXDSAFQ
Chondapi_DH2     1 HVVHGQVILPXXXXXDTAVR
Gynsun_DH4       1 HVLGGKKILPXXXXXEMTRF
Gynsun_DH7       1 HQIQGQKILPXXXXXEMARA
Gynsun_DH8       1 H--PEHKILPXXXXXEMVHV
Gynsun_DH9       1 HVIKGQIILPXXXXXHVIKG
Allov_DH4        1 HVLKGRKVLPXXXXXEMTRF
Allov_DH7        1 HVIRGEKILPXXXXXEMVRA
Allov_DH9        1 HTVQGRIILPXXXXXELVRA
```

DB shift domains

```
CorJ DH*         1 HTVLGQRVLLXXXXXDGVIV
DifK DH*         1 HLVFGKPALMXXXXXNSCYM
Pseubaet_DH11    1 HLVLGTPALMXXXXXNGCFF
Chromo_DH11      1 HKVLGKLALMXXXXXNSCFF
B8_DH11          1 HKVLGKPALMXXXXXNSCFF
Pseubaet_DH9     1 HRVNGQPALMXXXXXNSCYL
Chromo_DH9       1 HLVDGKPALMXXXXXNVCYQ
B8_DH9           1 HLVDGKPALMXXXXXNVCYQ
RhiE DH*         1 HQFNHRRILLXXXXXNSAFL
BaeR DH*         1 HQFSGEPVLVXXXXXNSAYL
```

Other DH domains

```
Pseubaet_DH10    1 HWVHGRPVFPXXXXXDGAAI
Chromo_DH10      1 HRVYGNPVFPXXXXXDAAAI
B8_DH10          1 HRVYGNPVFPXXXXXDAAAI
Pseudbae_DH8     1 HRVQGQAVLPXXXXXDGAAV
Chromo_DH8       1 HRVHGQRVLPXXXXXDGAAV
Gynsun_DH3b      1 HRVYGQRMIPXXXXXDFIYQ
Allov_DH3b       1 HHVHGHAILPXXXXXDIAYQ
```

PT-like DH domains

```
Pseubaet_DH13    1 HVGFYLEALYXXXXXDAALQ
Chromo_DH13      1 HVGFYLDAIYXXXXXDAALQ
B8_DH13          1 HVGFYLDAIYXXXXXDAALQ
Gynshun_DH12     1 HIGFYLETIYXXXXXDAVLQ
Allov_DH12       1 HIGFYLEALTXXXXXDAVVQ
Chondapi_DH11    1 HIGFYIETIYXXXXXDAVLQ
```

Figure S2. Extracted catalytic dyads from DH domains grouped as they cluster in the phylogenetic analysis; DB – double bond, DH – dehydratase domain.

Previously reported DB-shifting DH domains are labeled with an asterisk.

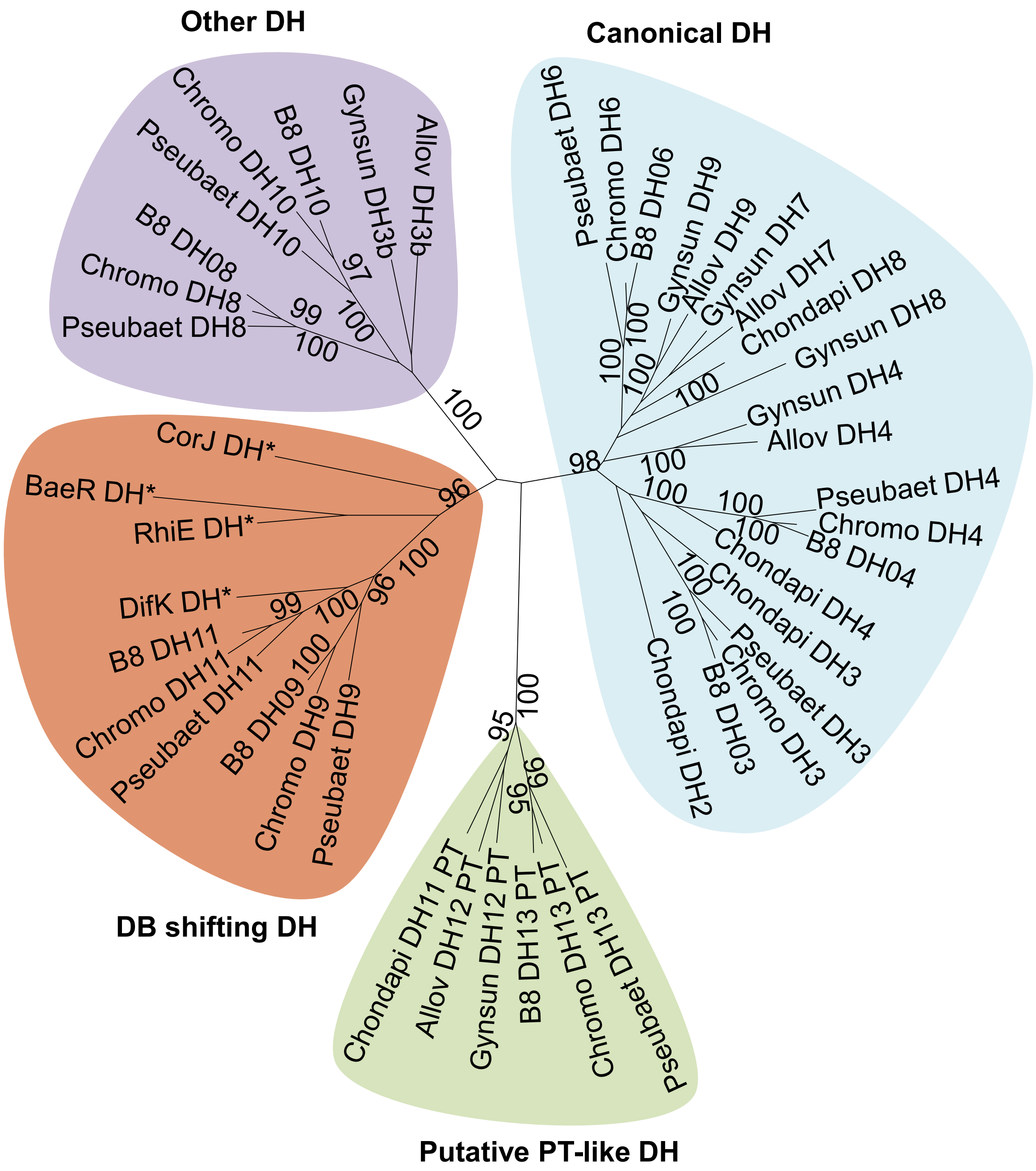

Figure S3. Phylogenetic analysis of DH domains from necroxime assembly line and related thiotemplate systems. DH domains cluster into canonical, inactive, double bond shifting (DB) and PT-like DH domains. Previously reported DB-shifting DH domains are labeled with an asterisk. B8 – *Burkholderia* sp. strain B8; Pseubaet – *Pseudomonas baetica*; Allov – *Allorhizobium vitis*; Chondapi – *Chrondromyces apiculatus*; Gynsun – *Gynuella sunshinyii*; Chromo – *Chromobacterium phragmitis* 112-1.

We excluded the amino acid sequences of B8 DH02 (dehydratase domain in module 2, B8), Pseubaet_DH2 and Chromo_DH2 from our phylogenetic analyses. DH2 showed a very distinct sequence that could not be matched with other DH amino acid sequences and thus forms its own clade.

Phylogeny of KS sequences: Ketosynthase (KS) sequences were taken from a previous study (in black)[15] and labeled according to their functions and specificities. Mega 6.06 was used to align the sequences (ClustalW)[16] and generate the phylogenetic tree (Maximum Likelihood Tree).[17] KS sequences from necroxime-like assembly lines (in blue) were assigned according to antiSMASH (Figure S4).

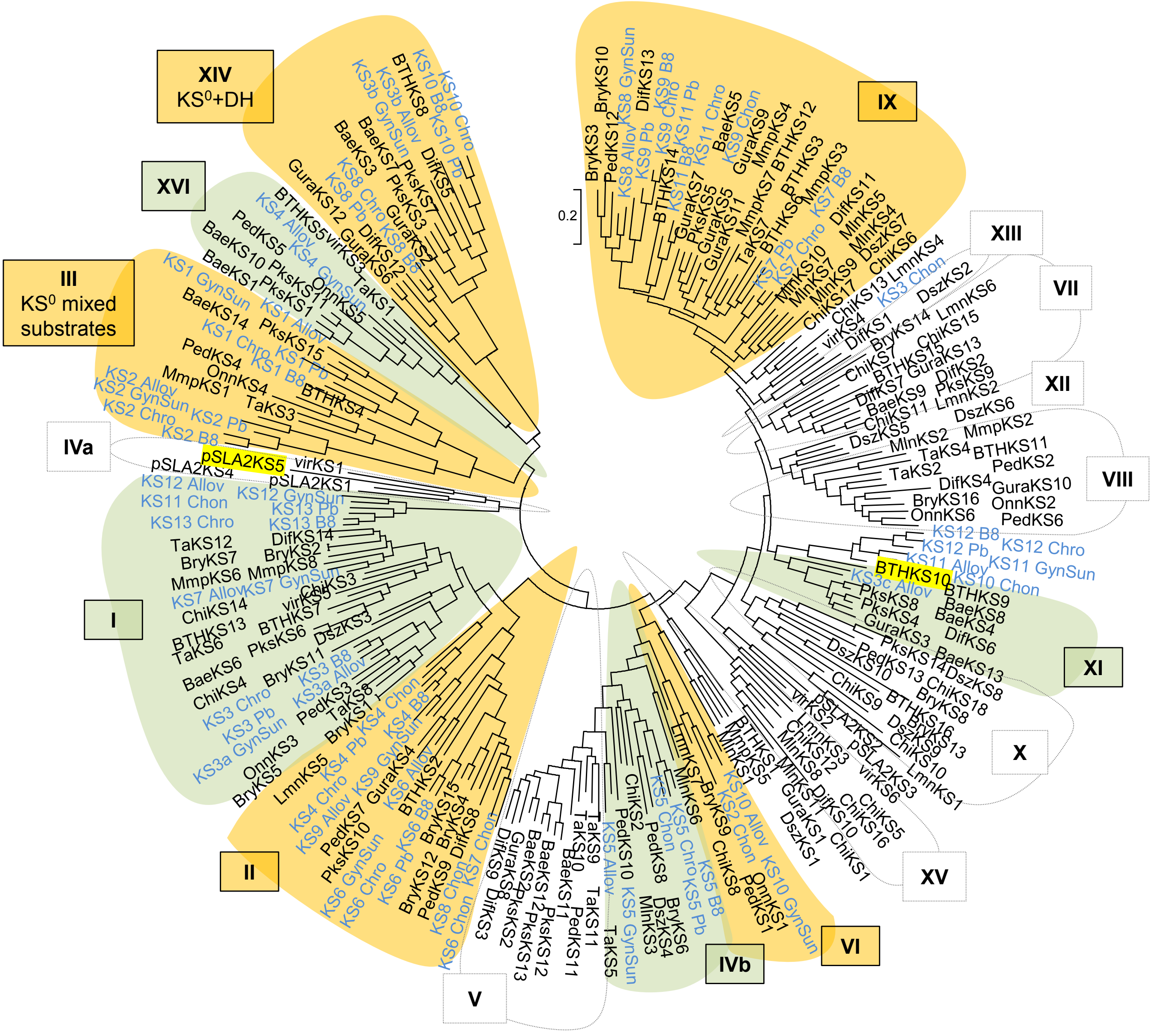

Figure S4. Phylogenetic analysis of substrate-specific ketosynthase domains. Group labels in boxes correspond to Nguyen *et al.* 2008. KS sequences of interest are colored accordingly from genomes of following organisms; B8 – *Burkholderia* sp. HKI404; Chro – *Chromobacterium phragmatis* IIBBL 112-1; Chon – *Chrondromyces apiculatus* DSM436; Pb – *Pseudomonas baetica* LMG 25716. GynSun – *Gyunella sunshinyii*. Allov – *Allorhizobium vitis.* Sequences highlighted in bright yellow represent exceptions. Numbering of KS according to module number.

Specificity of KR sequences: KR specificities were predicted according to Caffrey[18] (HXXXXXXD codes for D-β-OH). KR sequences were aligned using Mega6 and Maximum Likelihood Tree (Table S7).[16-17]

Table S7. Conserved amino acid residues coding for ketoreductase amino acid specificity.

Organism	Module number	KR specificity code	Prediction
Burkholderia sp. HKI-0404	3	**H** CAGAMA**N**AA	L
	4	**H** AAGGIR**D**SL	D
	5	**H** AAGVEE**G**RP	L
	6	**H** CAGVIE**D**NF	D
	7	**H** CAGLTS**A**DP	L
	9	**H** CAGVGD**A**NP	L
	12	**H** LAGVTT**D**AI	D
Chondromyces apiculatus DSM436	5	**H** AAGIAS**A**AS	L
	7	**H** AAGIES**A**RS	L
Pseudomonas baetica LMG 25716	5	**H** AAGVEE**G**DR	L
	6	**H** NAGLIA**D**DF	D
	9	**H** CAGLGD**Y**DH	L
Chromobacterium phragmitis IIBBL-112	5	**H** AAGAEE**G**GT	L
	9	**H** CAGMGD**A**NP	L
Rhizobium sp. BK-315	5	**H** AAGVES**D**KA	D
	8	**H** AALRDE**P**LD	L
Gynuella sunshinyii YC6258	5	**H** AAGVES**R**QP	L

Phylogeny of DH and PT (product template) domains: The amino acid sequences of characterized fungal PT domains (accession numbers given in the figure) were retrieved from the NCBI database and added to DH domains from known fungal polyketide pathways, fumonisin DH (ACB12550.1), bikaverin DH (CCT67991.1), isoterrein DH (EAU38790.1), terric acid DH (EAU32819.1), yanuthone-D DH (EHA22196.1), dehydrocurvularin DH (AGC95324.1), compactin DH (BAC20564.1), fujikurin DH (CCT72377.1), sordarin DH (BAV32159.1), citreoviridin DH (EAU29808.1), sorbicillin DH (CAP95405.1), andrastin-A DH (ART41209.1), and the necroxime DH domains. Sequences have been aligned using MAFFT 7.[12] Extensive gaps were manually deleted and the lengths of the sequences were adjusted for a more reliable phylogenetic analysis. Phylogenetic analysis was performed using IQ-tree.[13] Ultrafast bootstrapping (1,000 iterations) analysis was performed.
Neither the bacterial (necroxime DH domains) nor selected fungal DH domains tend to cluster with fungal PT domains, although they share similar folds and active site architectures. Instead, fungal and bacterial DH domains tend to form clusters next to each other. This could indicate that the putative PT-like DH domain (B8 DH13) in the necroxime PKS and fungal PT domains were subject to a congruent evolution that ultimately yielded a similar functionality.

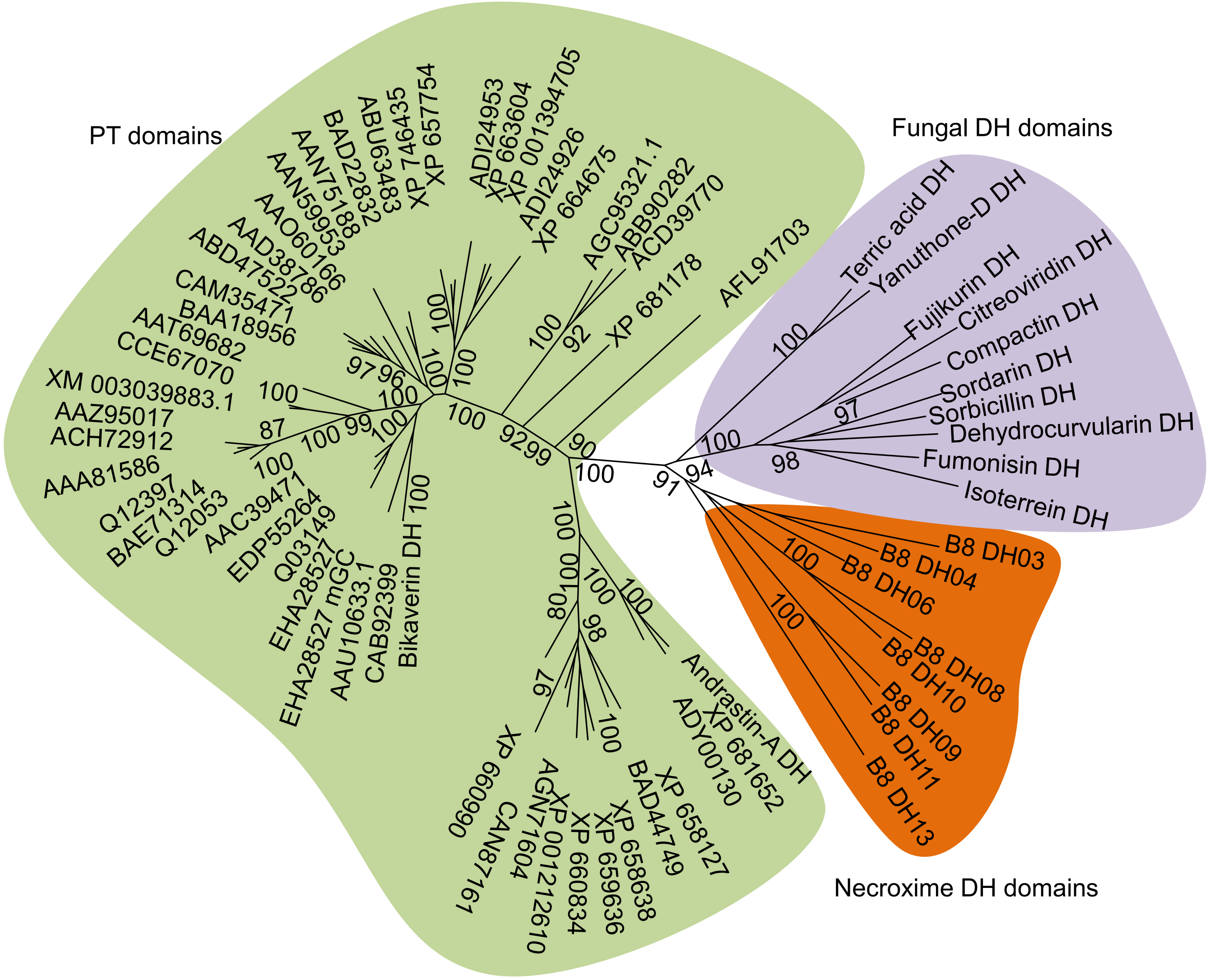

Figure S5. Phylogeny of fungal DH and PT (product template) domains and DH domains from the necroxime PKS.

General analytical methods

Analytical LC/MS: Exactive Orbitrap High Performance Benchtop LC-MS (Thermo Fisher Scientific) with an electron spray ion source and an Accela HPLC System, C18 column (Betasil C18 5 µm, 150 × 2.1 mm, Thermo Fisher Scientific), solvents: acetonitrile and water (both supplemented with 0.1% formic acid), flow rate: 0.2 mL min^{-1}; program: hold 1 min at 5% acetonitrile, 1–16 min 5–98% acetonitrile, hold 3 min 98% acetonitrile, 19–20 min 98% to 5% acetonitrile, hold 3 min at 5% acetonitrile.

MS/MS (tandem mass spectrometry): QExactive Orbitrap High Performance Benchtop LC/MS (ThermoFisher,) with an electron spray ion source and an Accela HPLC System, C18 column (Accucore C18 2.6 µm, 100 × 2.1 mm, Thermo Fisher Scientific) and the following solvent system: acetonitrile and water (both supplemented with 0.1% formic acid) at a flow rate of 0.2 mL min^{-1}; gradient: 0–10 min 5–98% acetonitrile, hold 4 min 98% acetonitrile, 14–14.1 min 98% to 5% acetonitrile, hold 6 min at 5% acetonitrile.

NMR: Bruker 600 MHz Avance III Ultra Shield (Bruker) and signals were referenced to the residual solvent signal at 2.50 ppm (^{1}H) or 39.51 ppm (^{13}C). ^{1}H 600 MHz, ^{13}C 150 MHz; NMR solvent (if not stated otherwise): DMSO-d_6.

Determination of the absolute configurations

Amino acid residue: The absolute configuration of lysine in necroxime A was determined by Marfey's method. Necroxime A (0.3 mg) was hydrolyzed by addition of 6 M HCl solution containing 0.05% phenol overnight at 105 °C. The mixture was concentrated under reduced pressure. Then, 100 µL 1 M $NaHCO_3$ solution and 50 µL L-FDAA (10 mg 1-fluoro-2,4-dinitrophenyl-5-L-alanine-amide in 1 mL acetone) were added and stirred at 50 °C for 1 h. Next, 50 µL 2 M HCl solution were added. The mixture was diluted by addition of 200 µL of 50% (*v*/*v*) acetonitrile.
Analytical HPLC conditions: Agilent Technologies 1100 Series HPLC with a UV detector G1315B and Gemini C18 column (phenomenex, 110 Å, 250 × 4.6 mm, 5 µm), flow rate: 0.5 mL min^{-1}, solvents: water and acetonitrile both supplemented with 0.1% trifluoroacetic acid, gradient: 0–5 min 0.5% acetonitrile, 5–35 min 0.5 to 100% acetonitrile.

Retention times of derivatized amino acid standards:
27.8 min for L-lysine, 29.2 min for D-lysine
Retention times of derivatized amino acid residue in necroxime A:
27.7 min (L-lysine)

Hydroxyl groups on the macrolactone ring: Mosher ester analysis was used to identify the stereochemistry of the OH-groups at C-10 and C-13 (Table S8). Necroxime C (1.5 mg) was dissolved in chloroform (0.3 mL). Next, water-free pyridine (16 µL) and MTPA-Cl (23 µL, either *R*-MTPA-Cl or *S*-MTPA-Cl) were added under an argon atmosphere. The mixture was stirred at room temperature for 2 h. The reaction was stopped by addition of water (1 mL) and subsequently extracted by addition of diethyl ether (three times 3 mL). The organic layers were combined, concentrated under reduced pressure and dissolved in methanol. Successful derivatization was monitored by LC/MS.
Preparative HPLC conditions: water/methanol, 5 mL min^{-1}, Synergi Fusion-RP column (phenomenex, 4 µm, 80 Å, 250 × 10 mm), 0–5 min 50% methanol, 5–10 min 50% to 100% methanol, 10–30 min 100% methanol. NMR spectra were monitored in CD_3OD.

Table S8. Results of Mosher ester analysis; selected ^{1}H NMR shifts of necroxime C (**3**) after derivatization with MTPA-Cl.

Signal	δ_H *R*-MTP-**3** [ppm]	δ_H *S*-MTP-**3** [ppm]	$\Delta(\delta_S - \delta_R)$
8	6.19	6.31	+0.12
9	5.59	5.74	+0.15
11	3.18	3.12	–0.06
12	2.84	3.07	+0.23
14a	2.23	2.14	–0.09
14b	1.96	1.87	–0.09
15	5.16	5.10	–0.06
17	5.08	5.00	–0.08
18	6.42	6.40	–0.02

Absolute configuration of 3-hydroxy butyric acid: 2 mg of necroxime A (**1**) was hydrolyzed with 6 M HCl solution (supplemented with 0.05% phenol) overnight at 105 °C. The mixture was concentrated under reduced pressure. Next, water-free pyridine (16 µL) and *S*-MTPA-Cl (23 µL) were added under an argon atmosphere. The mixture was stirred at room temperature for 2 h. The reaction was stopped by addition of water (1 mL) and subsequently extracted by addition diethyl ether (three times 3 mL). The organic layers were combined, concentrated under reduced pressure and dissolved in methanol. *R*-3-hydroxy butyric acid or *S*-3-hydroxy butyric acid (each 1 mg) were treated the same way. The derivatized samples were measured by LC/MS (QExactive).

Retention times of derivatized standards: 8.20 min for *R*-OH-butyric acid,
8.11 min for *S*-OH-butyric acid
Retention times of derivatized OH-butyric acid residue in necroxime A (**1**):
8.22 min (*R*-OH-butyric acid)

Absolute configuration of the epoxide functionality: A *cis* configuration for the epoxide was inferred from the coupling constant of H-12 and H-13 ($J_{11–12}$ 4.2 Hz) and a significant NOE signal. In addition, different NOEs (H-12 to H-15 and H-13; H-11 to H-10) suggest a similar orientation of the atoms on the macrocyclic ring. No NOE was observed for H_2-16 to H-12.

Determination of bioactivity

Cytotoxicity, antimicrobial, and antiproliferative profiling were conducted as previously described (Table S9).[19] The agar diffusion assay against *Sporobolomyces salmonicolor* was repeated with decreasing concentrations of necroxime A (Figure S5). The compounds were dissolved in DMSO for cytotoxicity assays (Table S10).

Table S9. Results from agar diffusion assays.

Strain	Necroxime A [1 mg mL^{-1}]	Ciprofloxacin [5 µg mL^{-1}]	Amphotericin B [10 µg mL^{-1}]	Methanol
Bacillus subtilis 6633 B1	no effect	29	n. d.	no effect
Staphylococcus aureus 511 B3	no effect	19	n. d.	no effect
Escherichia coli	no effect	24	n. d.	no effect
Pseudomonas aeruginosa SG 137 B7	no effect	25	n. d.	no effect
Pseudomonas aeruginosa K799/61 B9	no effect	27	n. d.	no effect
MRSA *Staphylococcus aureus* 134/94 R9	no effect	0	n. d.	no effect
VRSA *Enterococcus faecalis* 1528 R10	no effect	17	n. d.	no effect
Mycobacterium vaccae 10670 M4	no effect	20	n. d.	no effect
Sporobolomyces salmonicolor 549 H4	25	n. d.	19	10
Candida albicans H8	no effect	n. d.	21	no effect
Penicillium notatum JP36 P1	no effect	n. d.	20	10

Inhibition zones in mm. Test hole 9 mm; n. d., not determined.

Table S10. Cytotoxic and antiproliferative properties of the necroximes against several human cell lines.

	Antiproliferative effects [µM]					Cytotoxicity [µM]
Compound	HUVEC GI_{50}	K-562 GI_{50}	THP-1 GI_{50}	HEK-293 GI_{50}	Mono-Mac-6 GI_{50}	HeLa CC_{50}
Necroxime A (**1**)	0.60	4.91	8.63	44.64	8.78	1.93
Necroxime C (**3**)	1.53	1.09	n.d.	n.d.	n. d.	0.87
Necroxime D (**4**)	1.53	1.09	0.44	2.62	n. d.	1.09

n. d., not determined.

Table S10 continued. Cytotoxic and antiproliferative properties of the necroximes against several human cell lines.

	Antiproliferative effects [µM]	
Compound	Mono-Mac-6 GI_{50}	Raji GI_{50}
Necroxime A (**1**)	8.87	>75
Necroxime C (**3**)	n.d.	n.d.
Necroxime D (**4**)	n.d.	n.d.

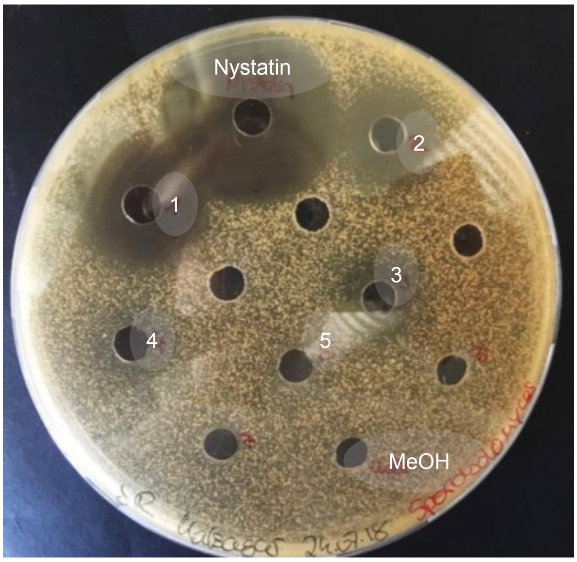

Figure S6. Agar diffusion assay of necroxime A against *S. salmonicolor*. Applied concentrations per mL: 1, 1 mg; 2, 0.5 mg; 3, 0.25 mg; 4, 100 µg; 5, 50 µg. Positive control: nystatin 50 mg. Solvent control: methanol.

NMR Tables

Table S11. NMR shifts of necroxime A (**1**). Measured in DMSO-d_6.

Position	δ_C [ppm]	δ_H [ppm]; Signal (*J* [Hz])
1	166.7	-
2	119.9	-
3	155.4	-
4	115.1	6.82; 1 H d (*8.6*)
5	130.4	7.20; 1 H t (*7.9*)
6	119.7	6.70; 1 H d (*7.4*)
7	135.8	-
8	131.5	6.55; 1 H d (*16.5*)
9	133.0	5.66; 1 H dd (*16.3; 9.1*)
10	69.4	4.52; 1 H d (*9.2*)
11	59.0	3.07; 1 H d (*4.1*)
12	55.6	2.88; 1 H dd (*8.6; 4.1*)
13	64.6	5.17; 1 H m
14	36.6	2.14; 1 H m
		1.83; 1 H m
15	70.9	5.17; 1 H m
16	34.6	2.38; 2 H t (*6.9*)
17	107.5	5.21; 1 H t (*7.7*)
18	125.7	6.74; 1 H dd (*13.7; 9.8*)
NH	-	10.27; 1 H d (*9.9*)
19	161.7	-
20	126.1	6.11; 1 H d (*11.5*)
21	133.4	6.51; 1 H t (*11.4*)
22	147.6	8.98; 1 H d (*10.3*)
23	61.9	3.86; 3 H s
24	171.4	-
25	51.7	4.17; 1 H m
26	30.1	1.69; 1 H m
		1.59; 1 H m
27	22.3	1.33; 2 H m
28	26.5	1.51; 2 H m
29	38.6	2.75; 2 H t (*7.5*)

Table S11 continued. NMR shifts of necroxime A (**1**). Measured in DMSO-d_6.

Position	δ_C [ppm]	δ_H [ppm]; Signal (*J* [Hz])
NH	-	8.18; 1 H d (*7.2*)
30	171.0	-
31	45.0	2.25; 1 H dd (*13.8; 6.9*)
		2.12; 1 H*
32	63.7	3.92; 1 H sext (*6.4*)
33	23.3	1.05; 3 H d (*6.1*)
3-OH	-	9.92; 1 H br
10-OH	-	5.52; 1 H br
34-OH	-	4.60; 1 H br

* Overlapping signals

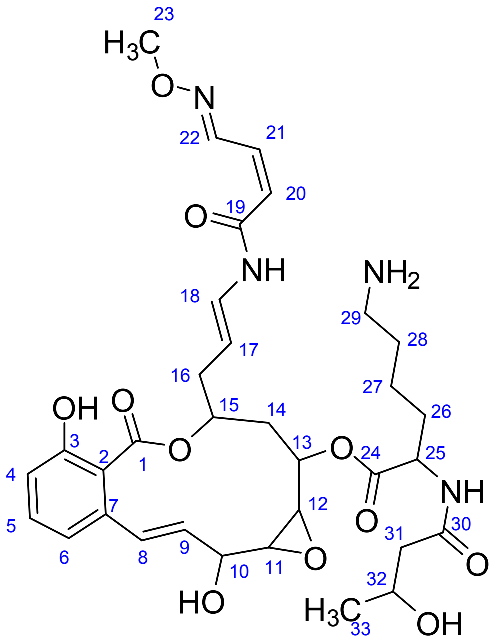

Figure S7. Structure of necroxime A with NMR assignments.

Table S12. NMR shifts of necroxime B (**2**). Measured in DMSO-d_6.

Position	δ_C [ppm]	δ_H [ppm]; Signal (*J* [Hz])
1	166.7	-
2	119.9	-
3	155.4	-
4	115.1	6.82; 1 H d (*8.9*)
5	130.4	7.20; 1 H t (*8.0*)
6	119.7	6.70; 1 H d (*7.2*)
7	135.8	-
8	131.5	6.55; 1 H d (*17.8*)
9	133.0	5.66; 1 H dd (*17.5; 9.2*)
10	69.4	4.52; 1 H d (*9.5*)
11	59.0	3.07; 1 H d (*3.8*)
12	55.6	2.87; 1 H dd (*9.0; 4.1*)
13	64.6	5.18; 1 H m
14	36.8	2.14; 1 H m
		1.80; 1 H m
15	70.9	5.18; 1 H m
16	34.6	2.38; 2 H m
17	107.5	5.21; 1 H m
18	125.7	6.73; 1 H dd (*14.8; 10.6*)
NH	-	10.25; 1 H d (*11.0*)
19	161.7	-
20	126.1	6.11; 1 H d (*11.3*)
21	133.4	6.51; 1 H t (*11.2*)
22	147.6	8.96; 1 H d (*10.6*)
23	61.9	3.85; 3 H s
24	171.5	-
25	51.7	4.17; 1 H m
26	30.1	1.69; 1 H m
		1.59; 1 H m
27	22.4	1.32; 2 H m
28	26.5	1.51; 2 H m
29	38.6	2.74; 2 H m

Table S12 continued. NMR shifts of necroxime B (**2**). Measured in DMSO-d_6.

Position	δ_C [ppm]	δ_H [ppm]; Signal (J [Hz])
NH	-	8.16; 1 H d (*7.7*)
30	172.6	-
31	36.9	2.06; 2 H t (*7.5*)
32[a]	18.7	1.48; 2 H quin (*7.4*)
33	13.6	0.82; 3 H t (*7.4*)
3-OH	-	9.92; 1 H br

[a] In necroxime B (compared to A) the 3-hydroxy butyric acid is exchanged with butyric acid.

Table S13. NMR shifts of necroxime C (**3**). Measured in DMSO-d_6.

Position	δ_C [ppm]	δ_H [ppm]; Signal (*J* [Hz])
1	167.0	-
2	120.3	-
3	154.9	-
4	119.5	6.81; 1 H d (*8.0*)
5	130.0	7.20; 1 H t (*7.8*)
6	120.2	6.69; 1 H d (*7.4*)
7	135.8	-
8	131.1	6.51; 1 H d (*16.5*)
9	133.7	5.69; 1 H dd (*9.2; 16.4*)
10	69.5	4.49; 1 H dd (*4.2; 9.2*)
11	58.6	3.07; 1 H d (*4.3*)
12	58.3	2.76; 1 H dd (*4.2; 8.7*)
13	59.9	3.97; 1 H m
14	40.3	1.87; 1 H m
		1.77; 1 H m
15	71.5	5.15; 1 H m
16	34.7	2.35; 2 H t (*6.8*)
17	107.3	5.24; 1 H dt (*8.0; 14.5*)
18	125.5	6.76; 1 H dd (*10.3; 14.5*)
NH	-	10.23; 1 H d (*10.2*)
19	161.3	-
20	130.5	6.39; 1 H d (*15.3*)
21	132.8	7.00; 1 H dd (*10.2; 15.5*)
22	148.9	8.05; 1 H d (*10.6*)
23	62.0	3.88; 3 H s
3-OH	-	9.86; 1 H br
10-OH	-	5.46; 1 H br

Table S14. NMR shifts of necroxime D (**4**). Measured in DMSO-d_6.

Position	δ_C [ppm]	δ_H [ppm]; Signal (J [Hz])
1	166.8	-
2	120.2	-
3	154.8	-
4	114.8	6.80; 1 H d (*8.2*)
5	130.0	7.18; 1 H t (*8.0*)
6	119.5	6.68; 1 H d (*7.5*)
7	135.8	-
8	131.1	6.49; 1 H d (*16.2*)
9	133.6	5.67; 1 H dd (*9.2; 16.9*)
10	69.5	4.47; 1 H dd (*4.7; 9.1*)
11	58.5	3.05; 1 H d (*4.3*)
12	58.3	2.74; 1 H dd (*4.2; 8.5*)
13	59.8	3.96; 1 H m
14	40.3	1.86; 1 H m
		1.74; 1 H m
15	71.5	5.14; 1 H m
16	34.9	2.34; 2 H t (*7.1*)
17	107.8	5.20; 1 H dt (*7.0; 14.2*)
18	125.5	6.72; 1 H dd (*10.4; 14.4*)
NH	-	10.23; 1 H d (*10.3*)
19	161.6	-
20	126.2	6.10; 1 H d (*11.6*)
21	133.3	6.52; 1 H dd (*10.2; 11.3*)
22	147.5	8.97; 1 H d (*10.5*)
23	61.9	3.85; 3 H s
3-OH	-	9.88; 1 H br
10-OH	-	5.44; 1 H d (*5.3*)
13-OH	-	5.03; 1 H d (*5.4*)

MS/MS spectra

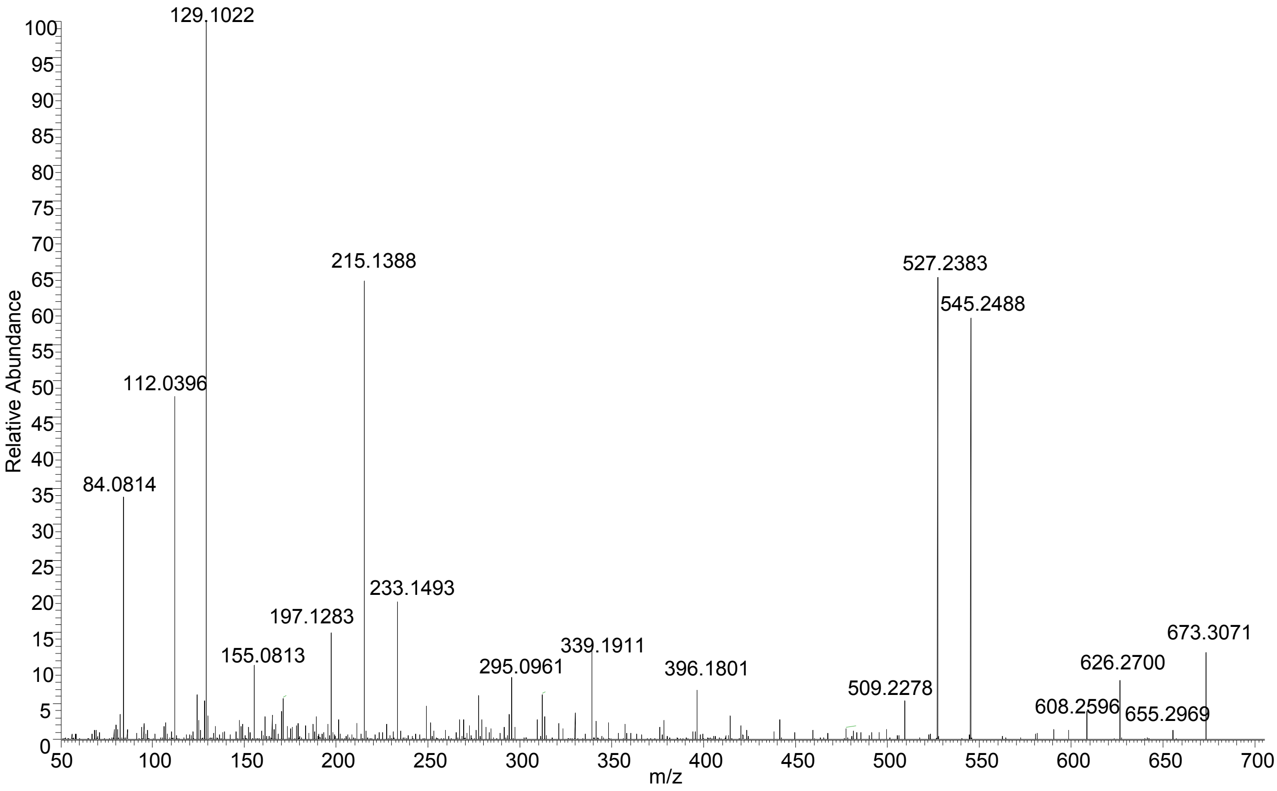

Figure S8. MS/MS fragmentation pattern of *m/z* 673.3071 $[M+H]^+$, necroxime A (**1**).

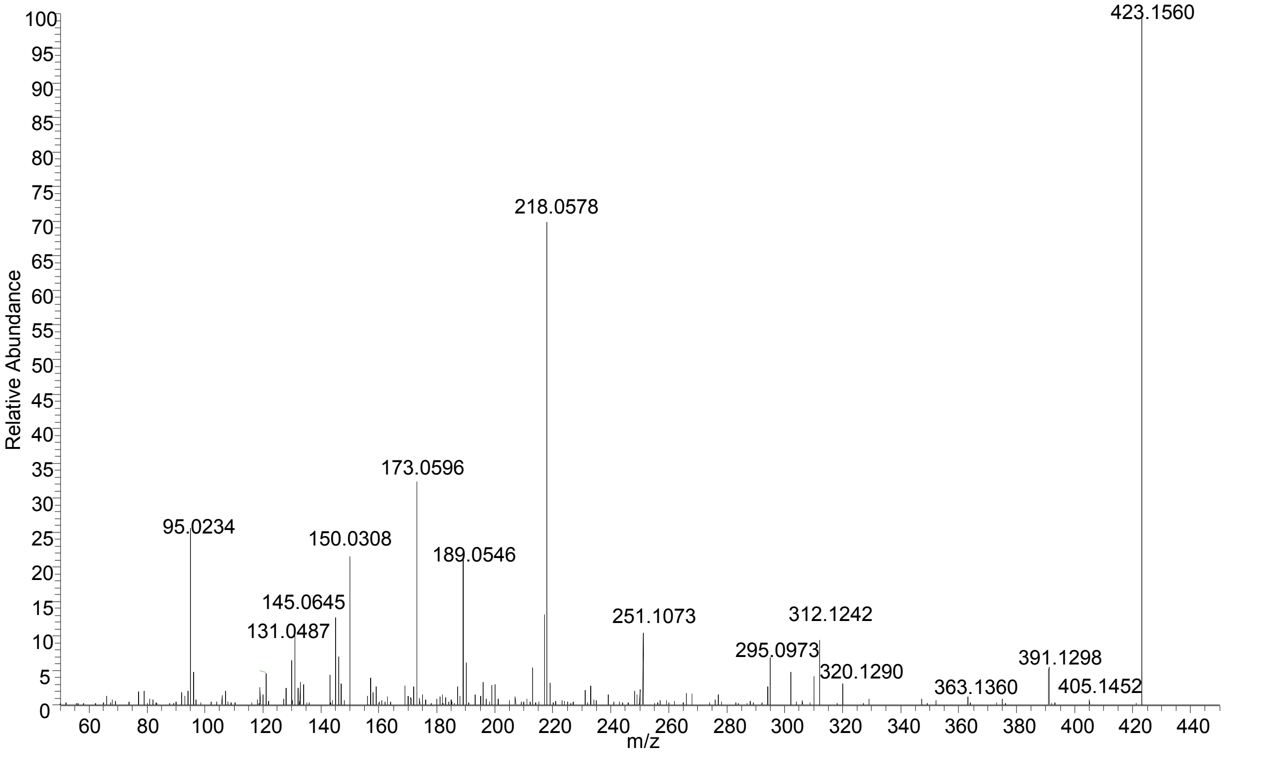

Figure S9. MS/MS fragmentation pattern of *m/z* 423.1560 $[M{-}H]^-$ in *P. baetica* extracts, oximidine II/III (**11**).

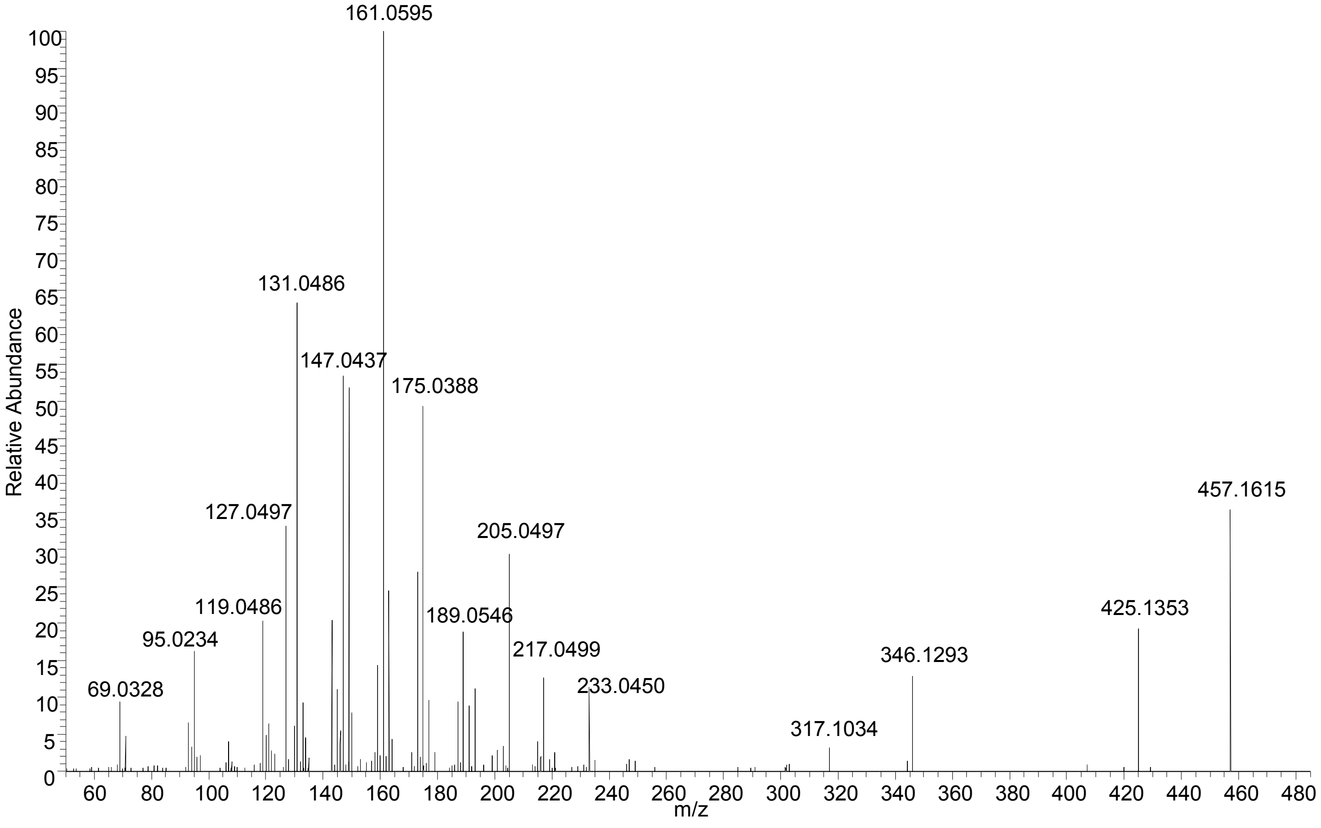

Figure S10. MS/MS fragmentation pattern of *m/z* 457.1615 $[M–H]^-$ in *Burkholderia* sp. HKI-0404 extracts (**3**).

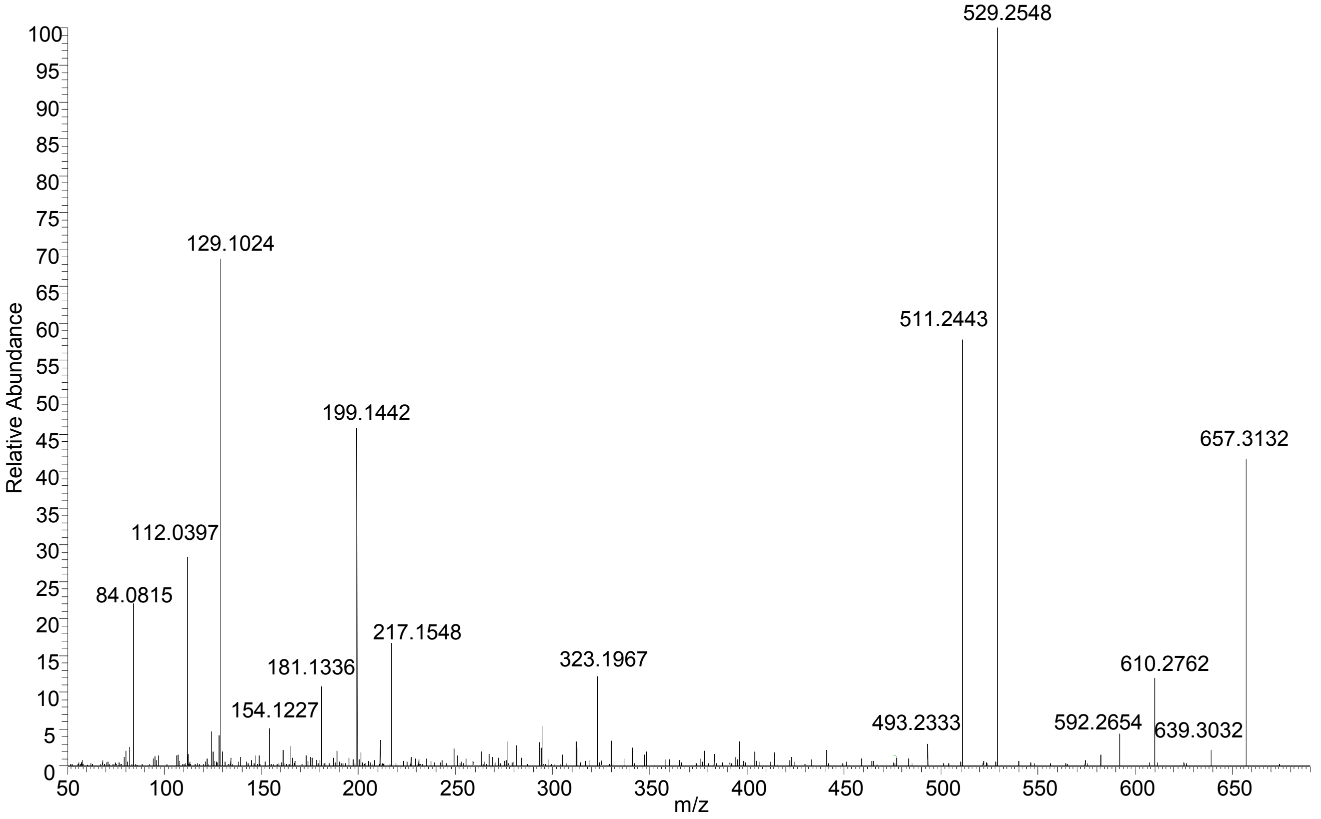

Figure S11. MS/MS fragmentation pattern of m/z 657.3132 $[M+H]^+$ in *Burkholderia* sp. HKI-0404 extracts (**2**).

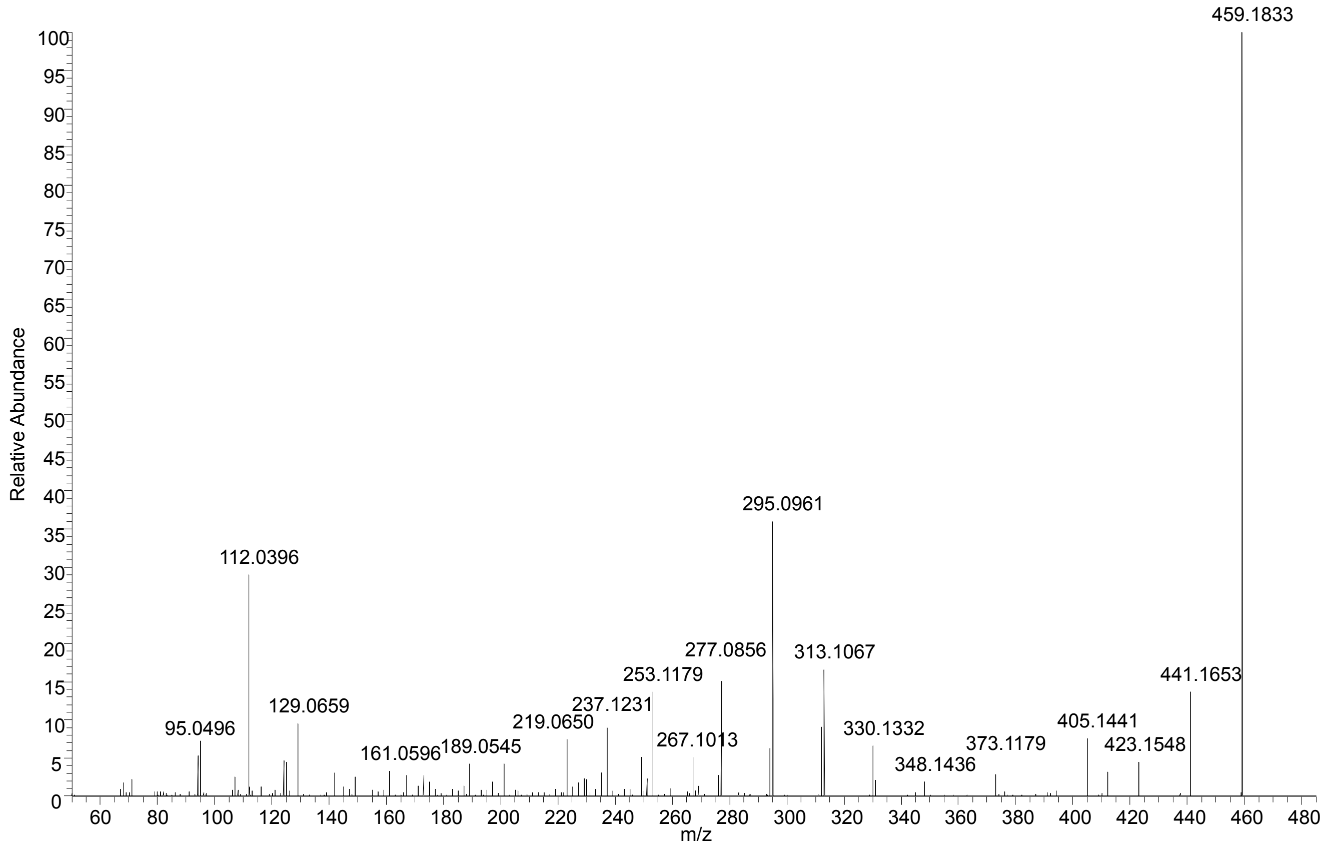

Figure S12. MS/MS fragmentation pattern of *m/z* 459.1833 $[M+H]^+$ in *Burkholderia* sp. HKI-0404 extracts (**3**).

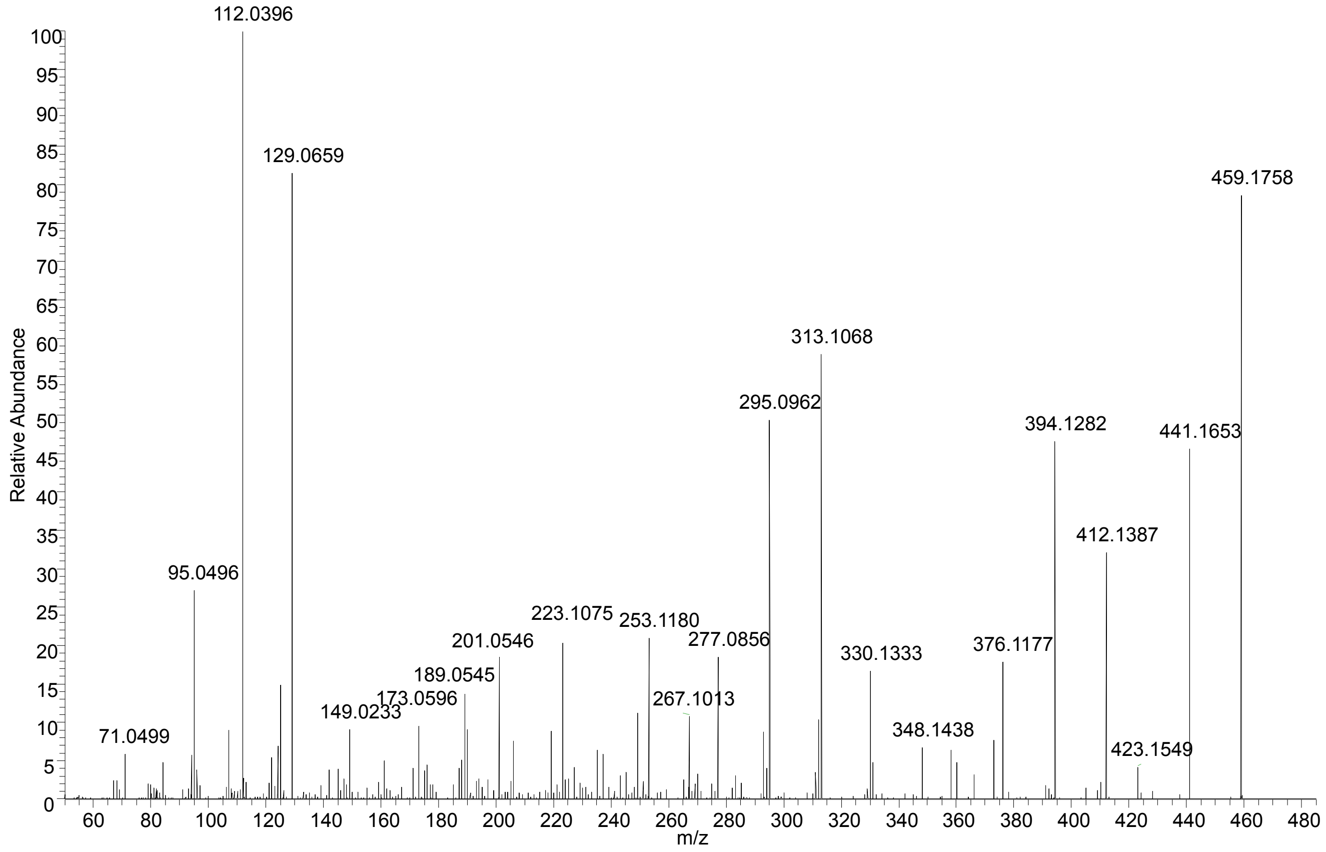

Figure S13. MS/MS fragmentation pattern of *m/z* 459.1758 $[M+H]^+$ in *Burkholderia* sp. HKI-0404 extracts (**4**).

NMR spectra

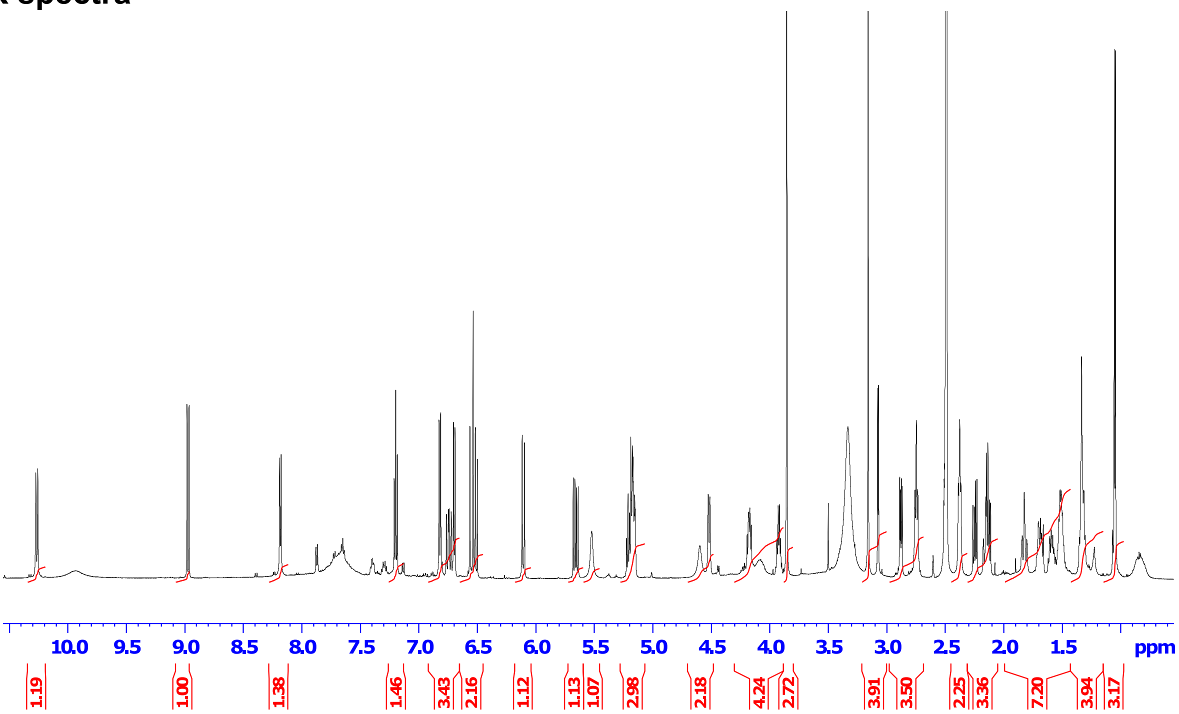

Figure S14. ^{1}H NMR spectrum of necroxime A.

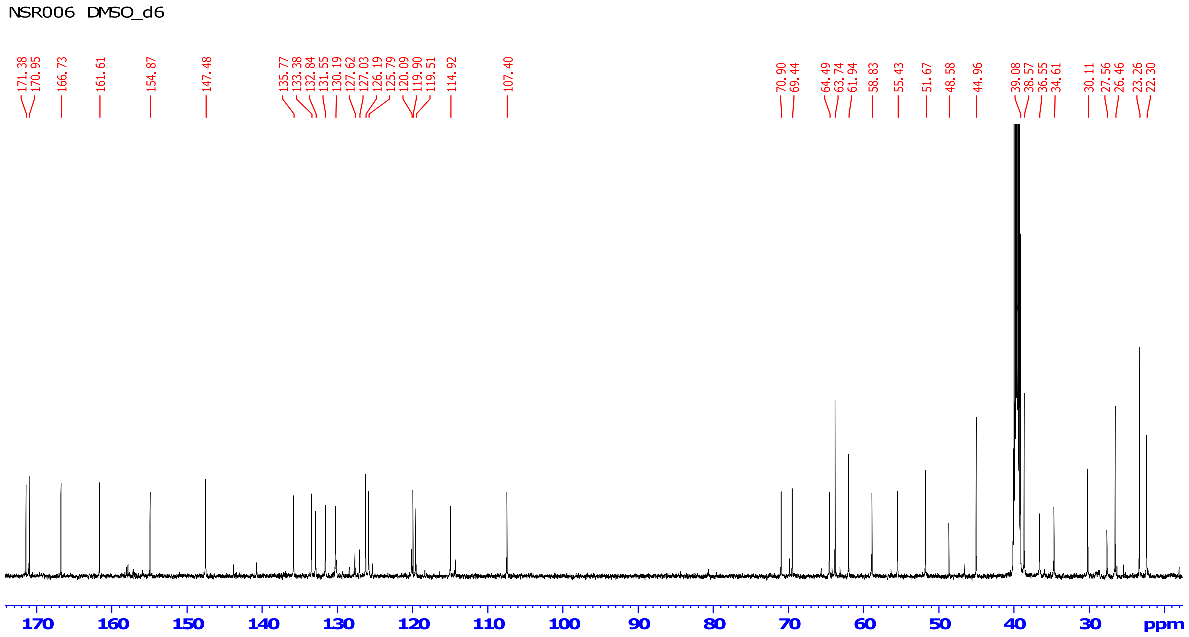

Figure S15. ^{13}C NMR spectrum of necroxime A.

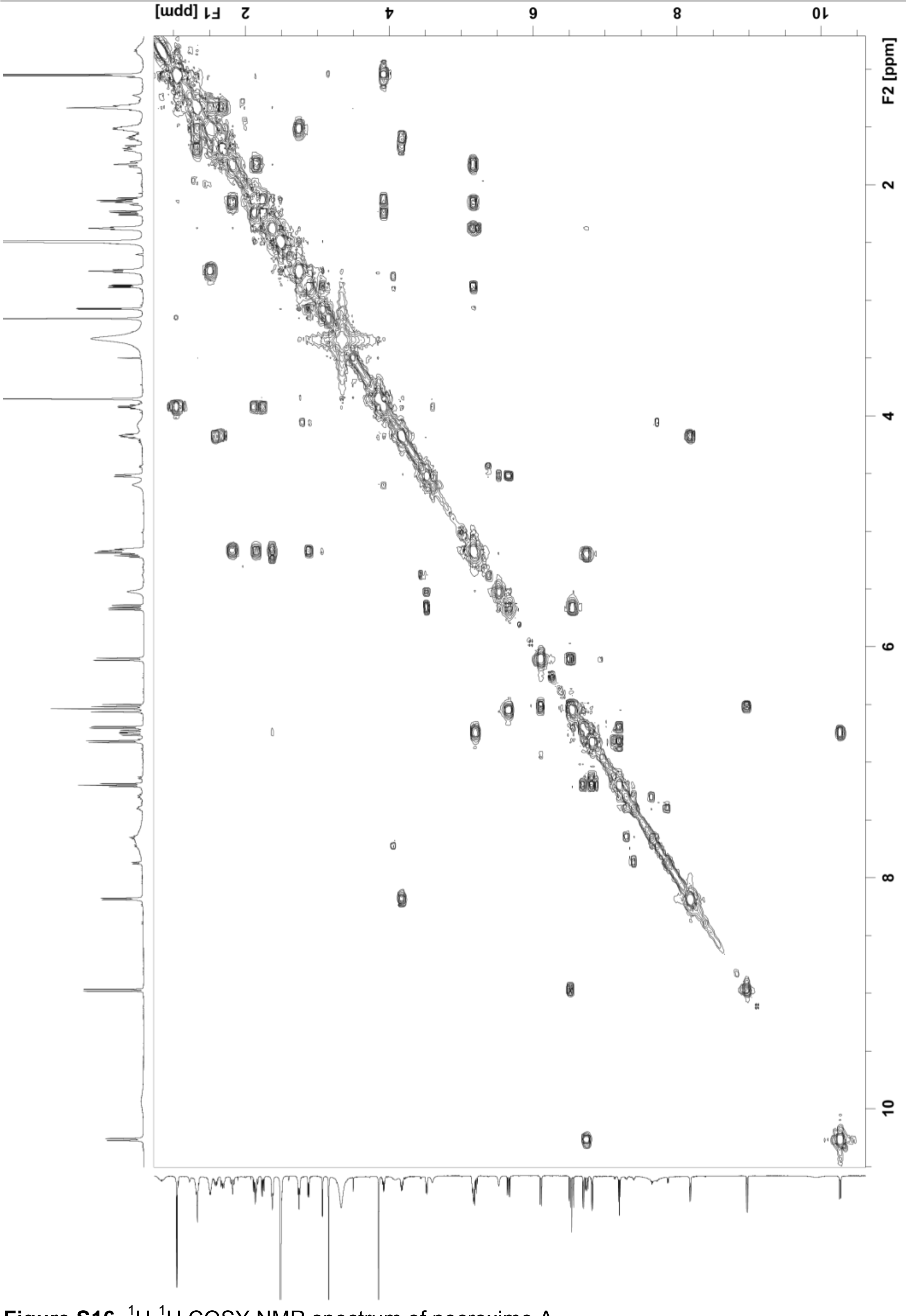

Figure S16. ^{1}H-^{1}H COSY NMR spectrum of necroxime A.

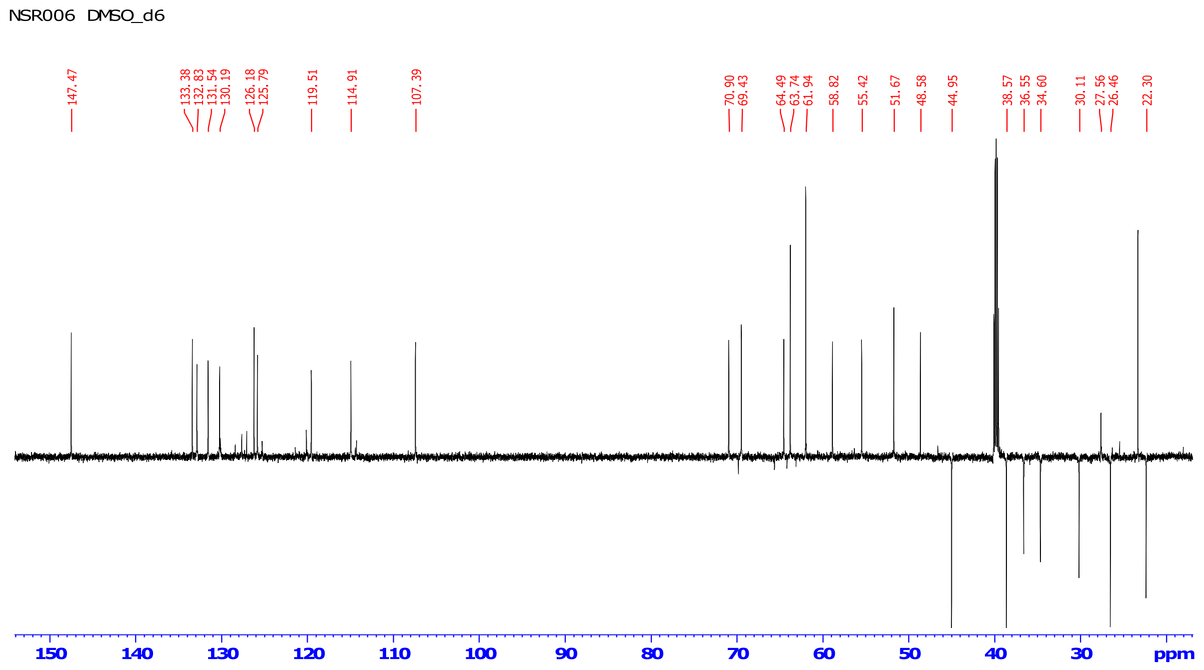

Figure S17. DEPT-135 NMR spectrum of necroxime A.

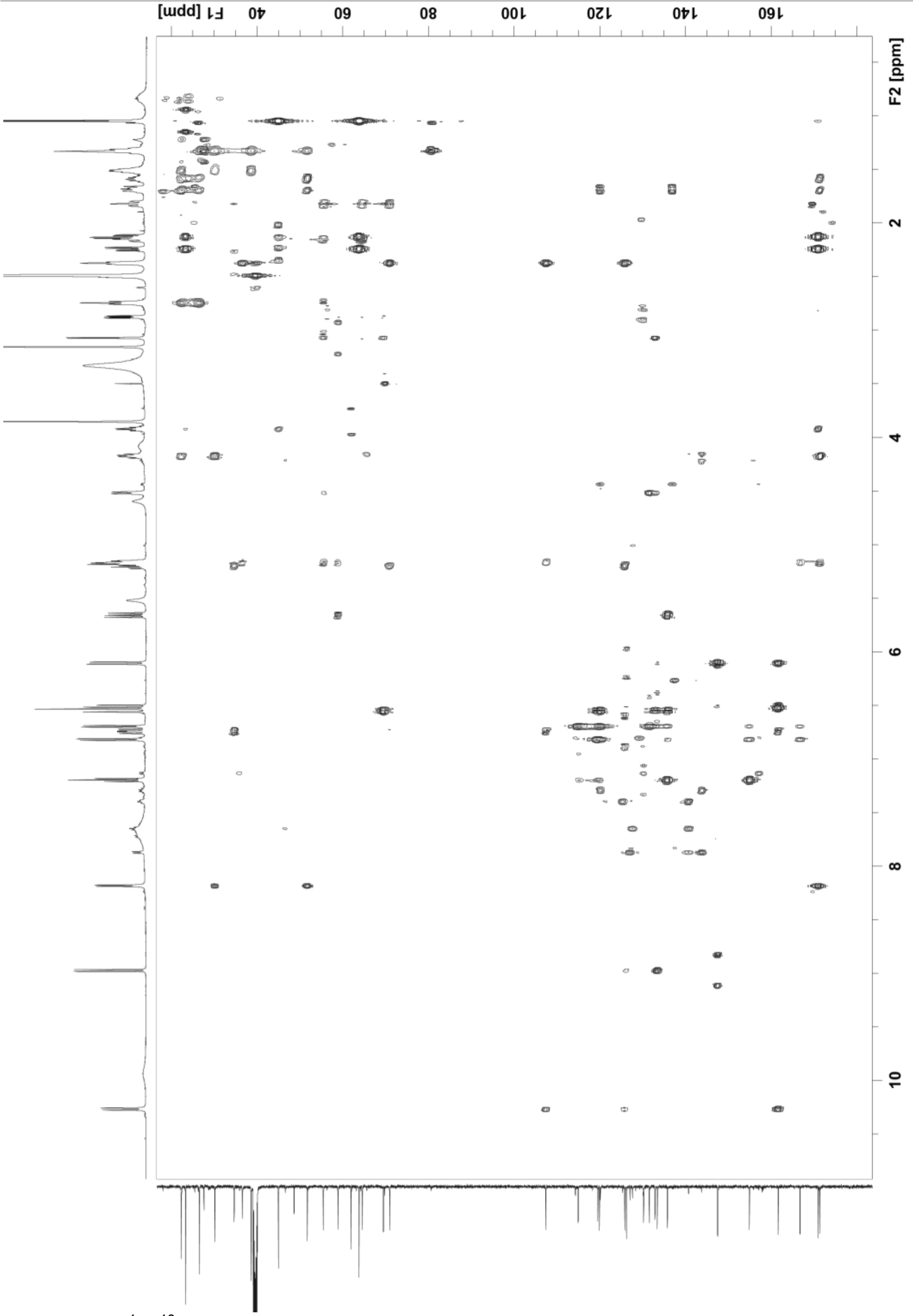

Figure S18. ^{1}H-^{13}C HMBC NMR spectrum of necroxime A.

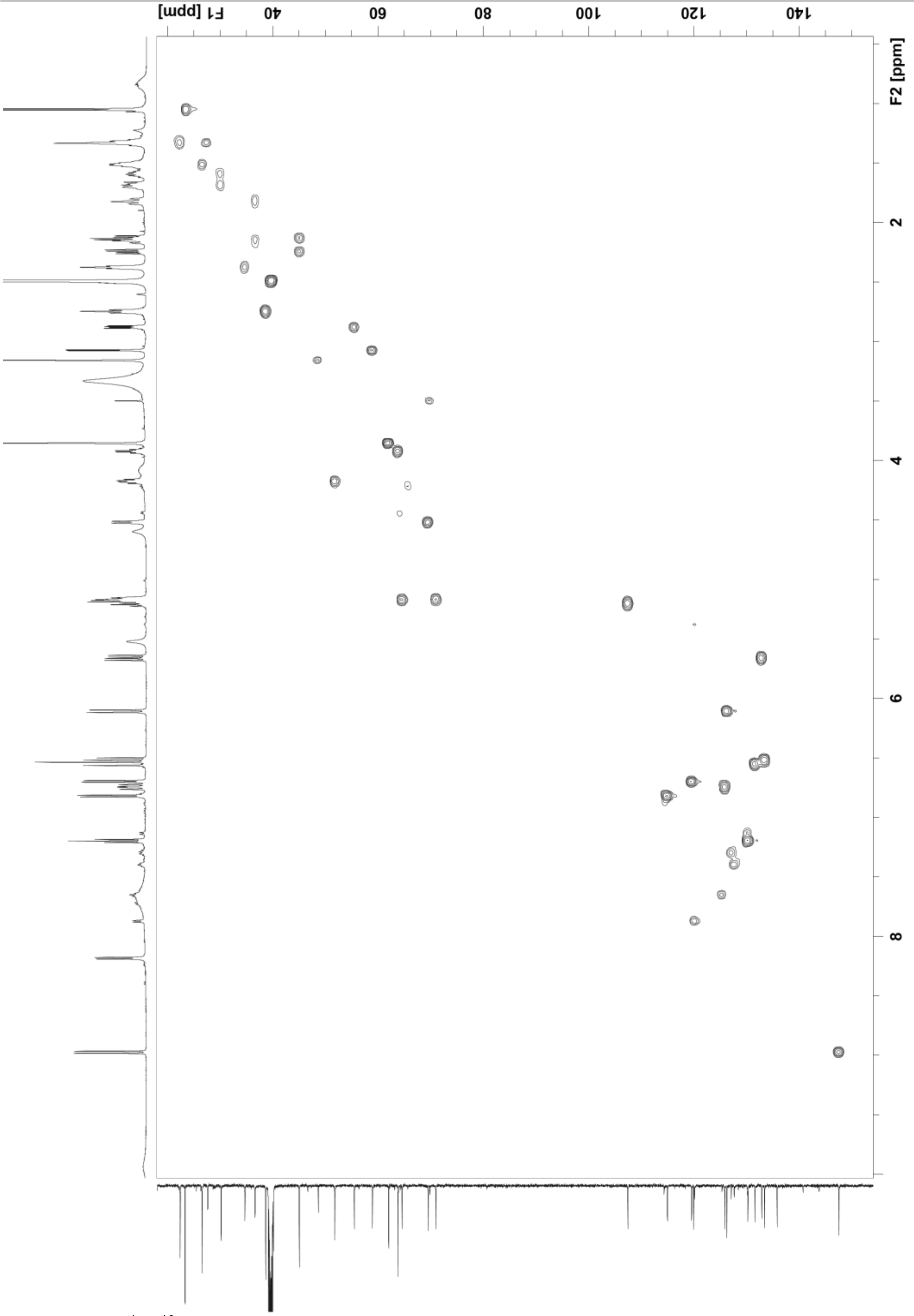

Figure S19. ^{1}H-^{13}C HSQC NMR spectrum of necroxime A.

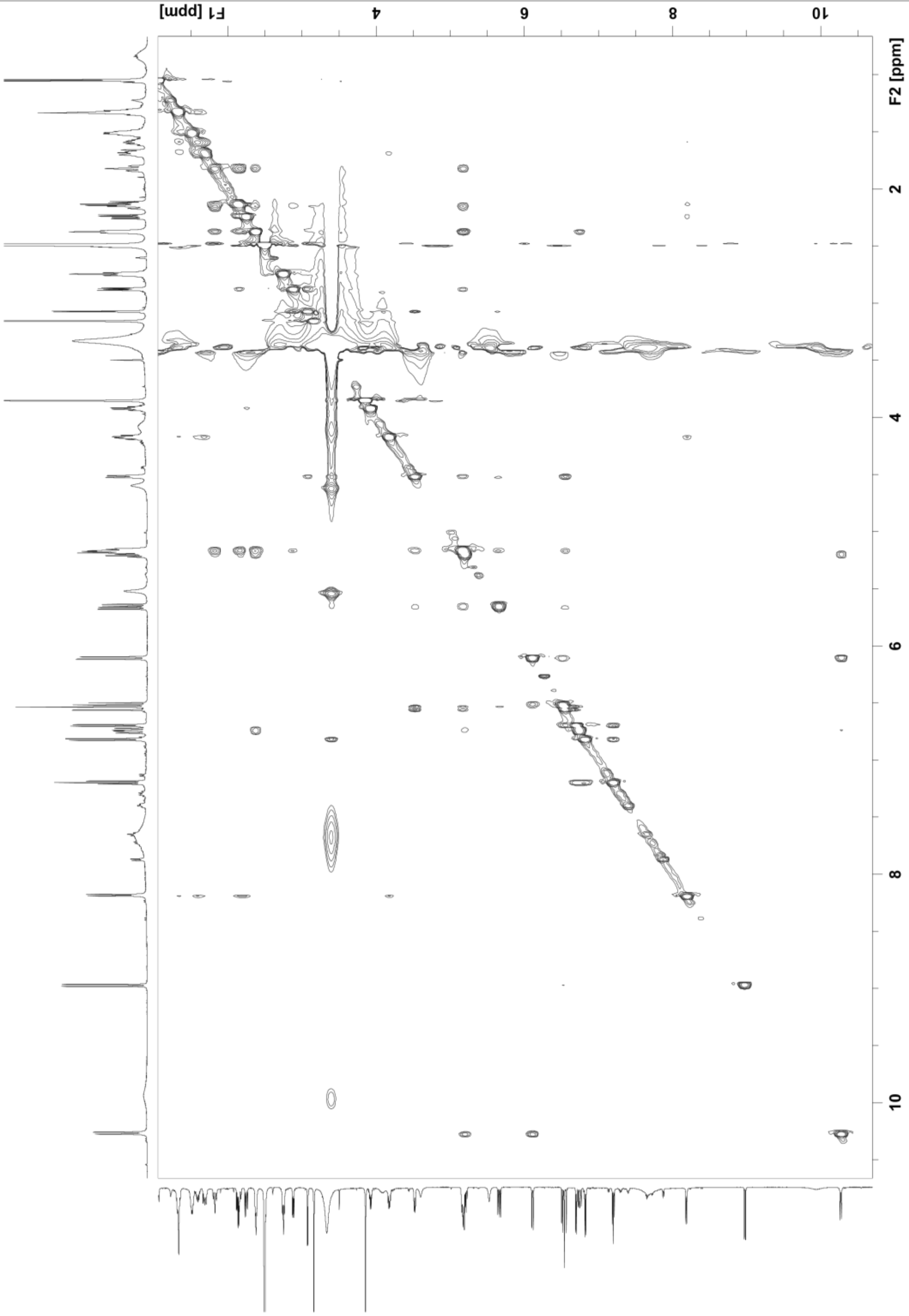

Figure S20. ^{1}H-^{1}H NOESY NMR spectrum of necroxime A.

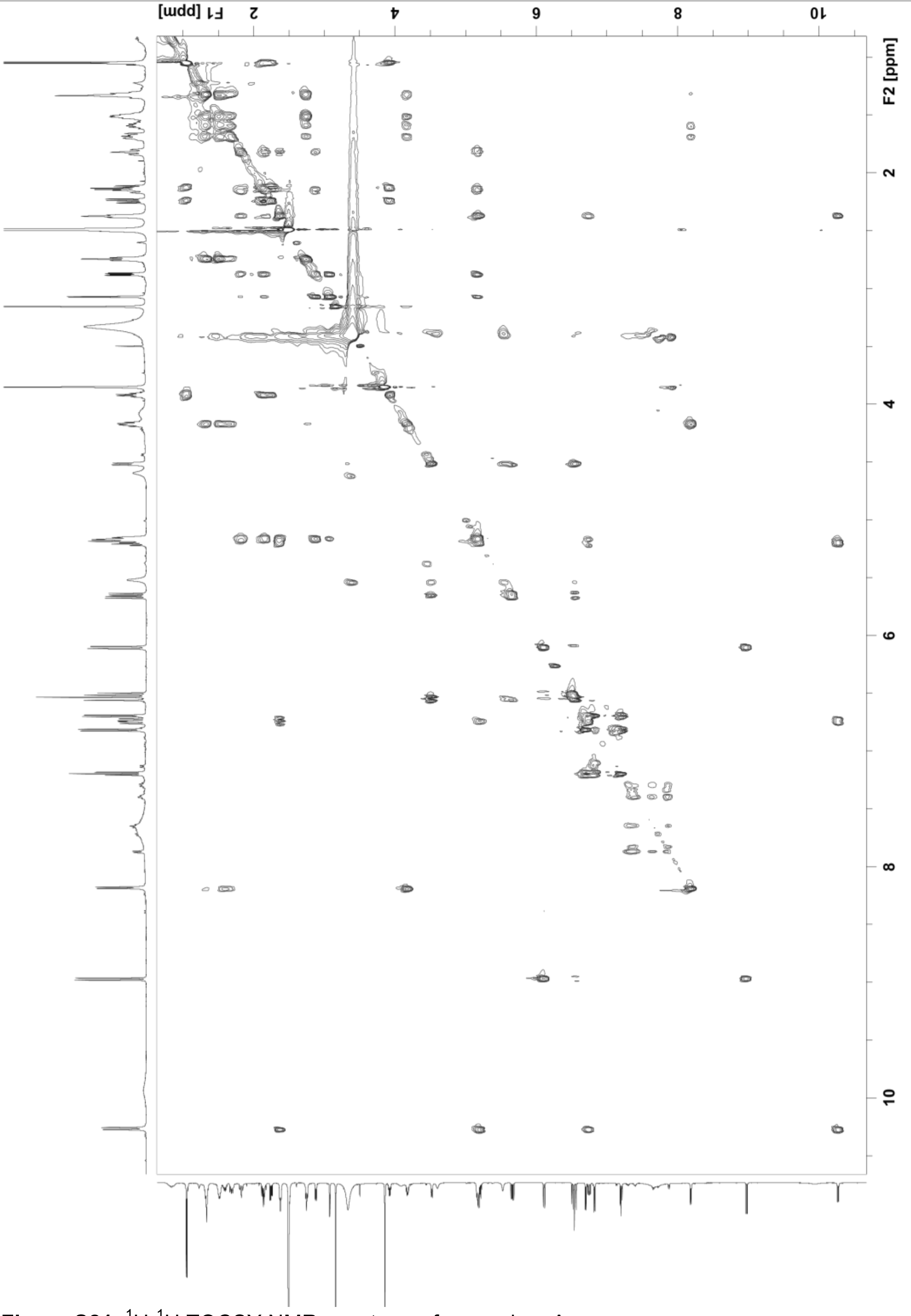

Figure S21. ^{1}H-^{1}H TOCSY NMR spectrum of necroxime A.

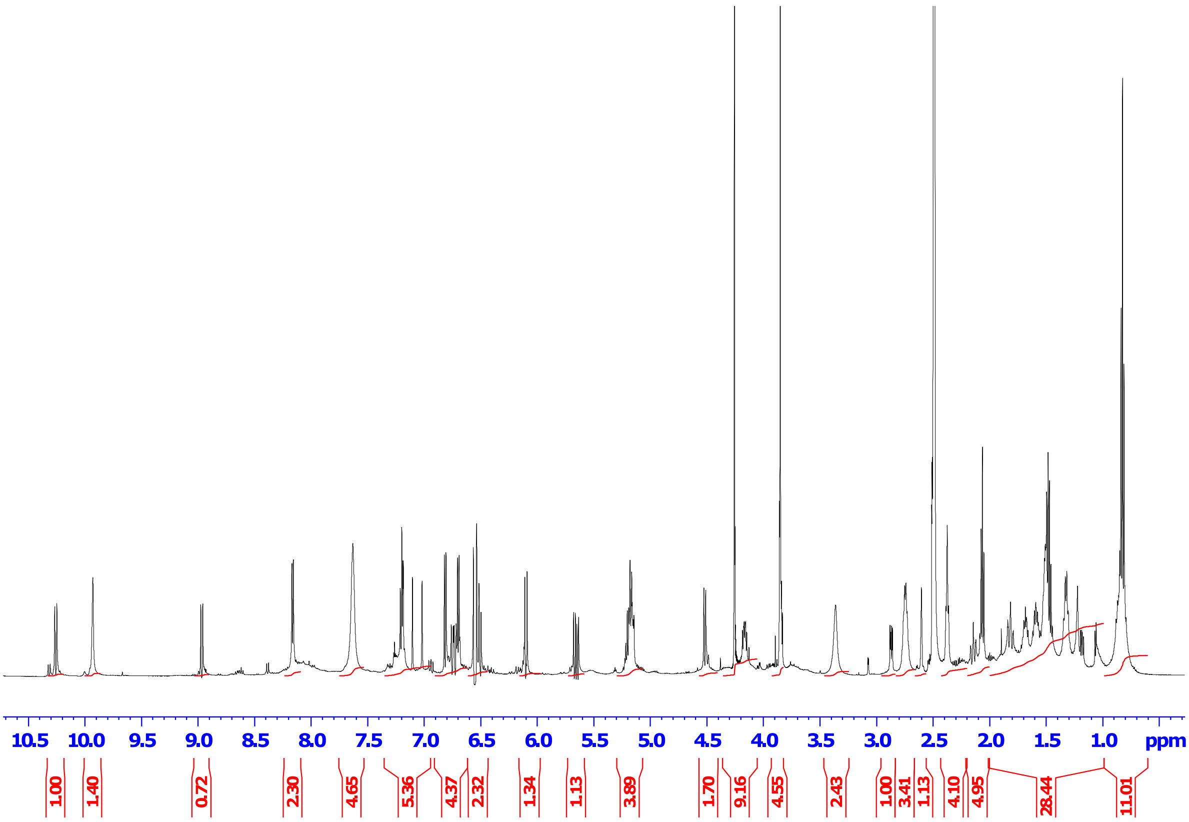

Figure S22. ¹H NMR spectrum of necroxime B.

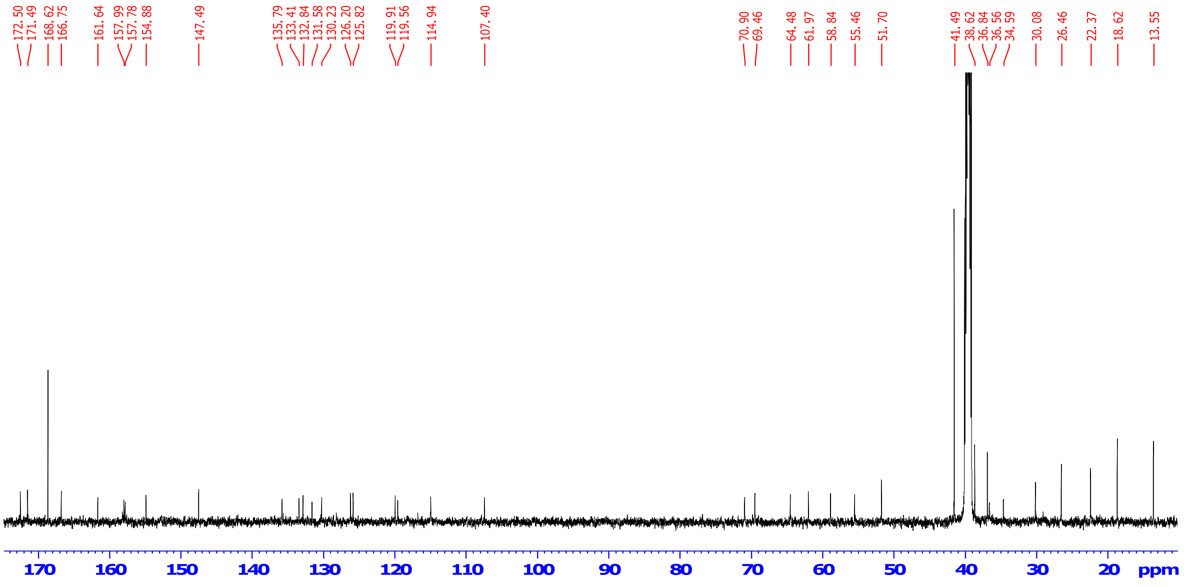

Figure S23. ^{13}C NMR spectrum of necroxime B.

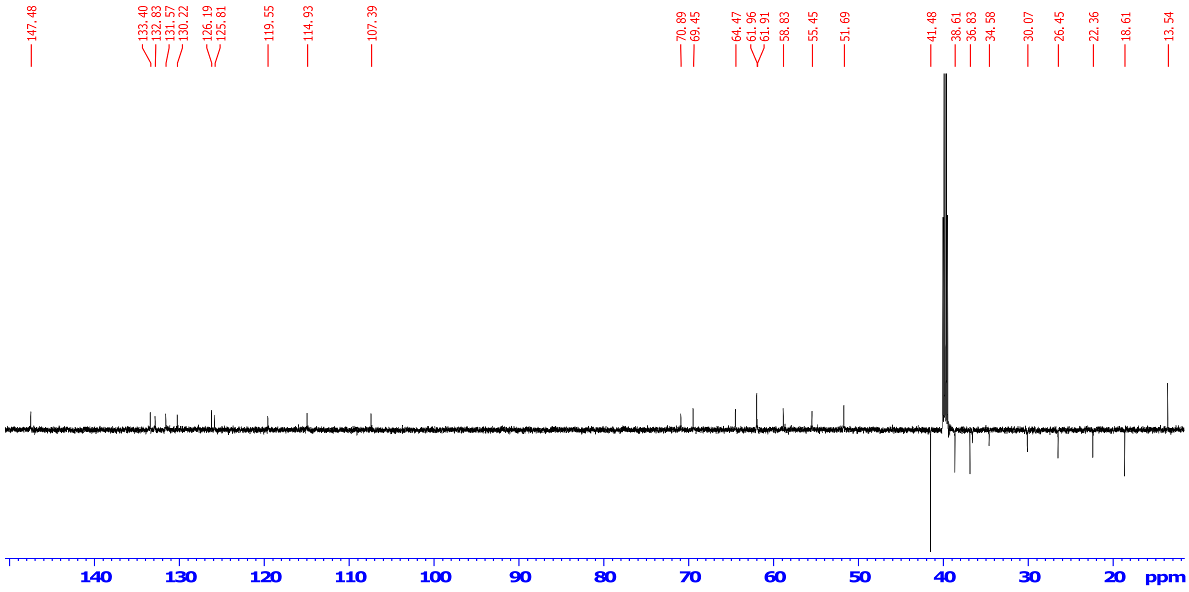

Figure S24. DEPT-135 NMR spectrum of necroxime B.

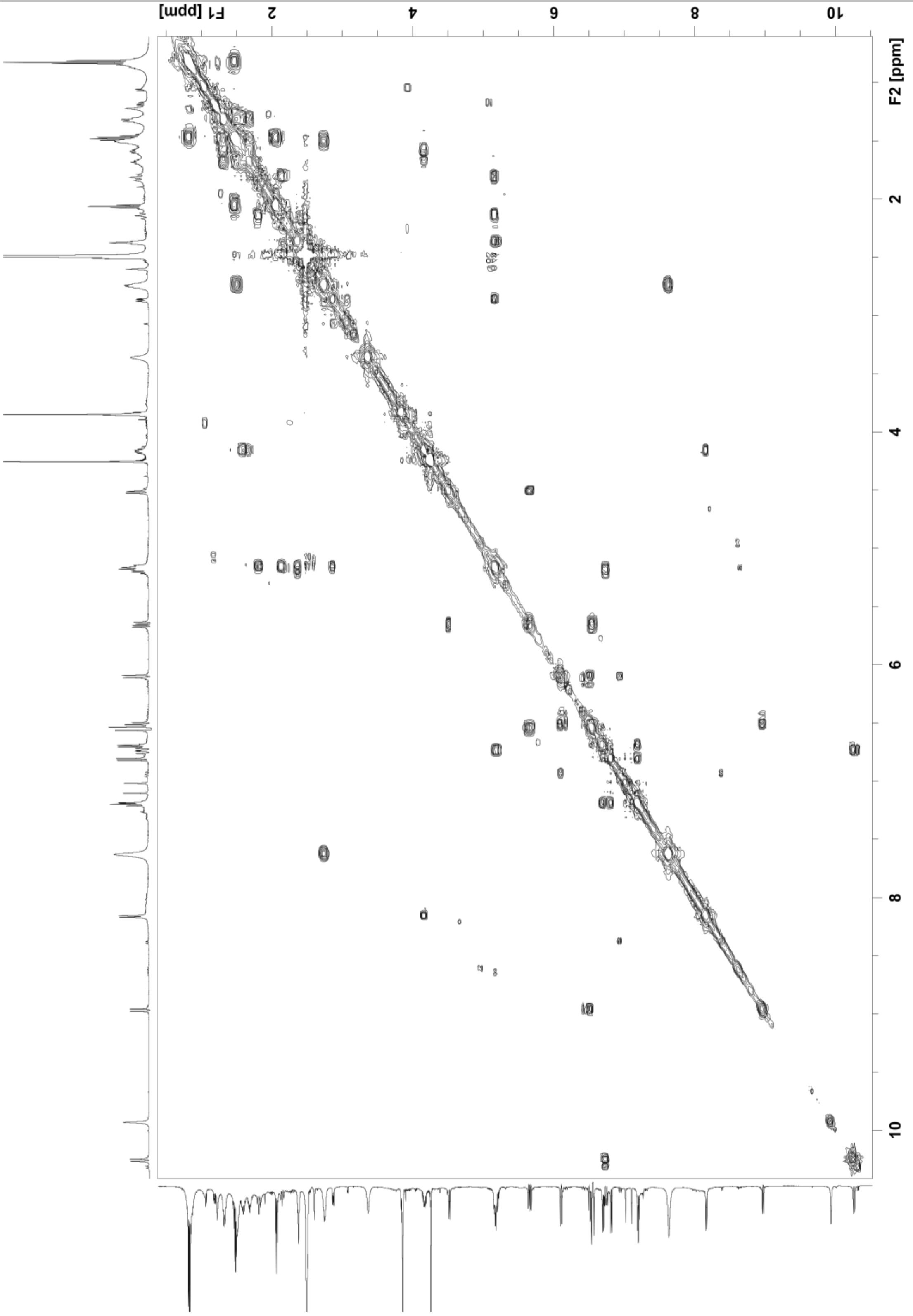

Figure S25. $^{1}H,^{1}H$ COSY NMR spectrum of necroxime B.

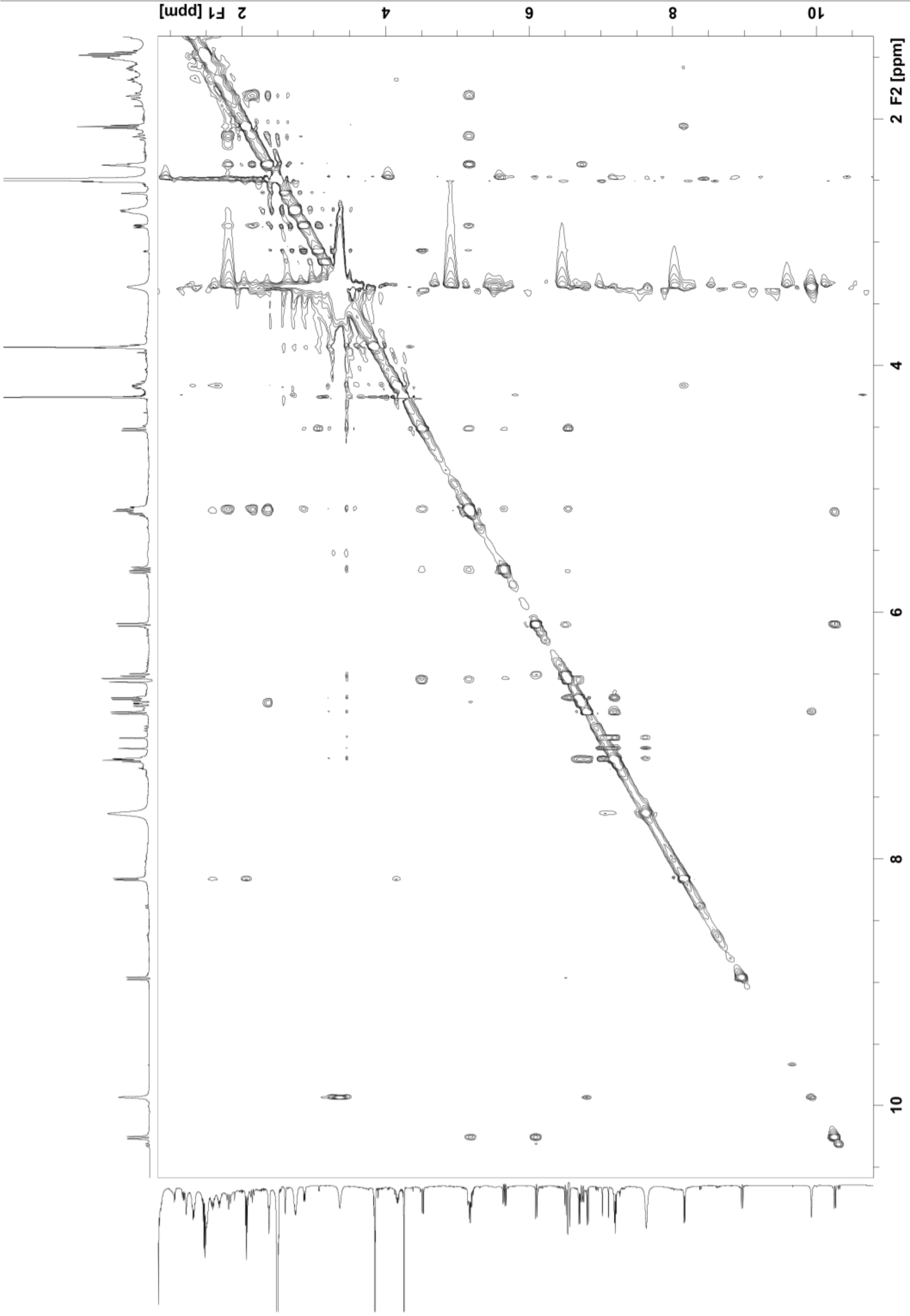

Figure S26. $^{1}H,^{1}H$ NOESY NMR spectrum of necroxime B.

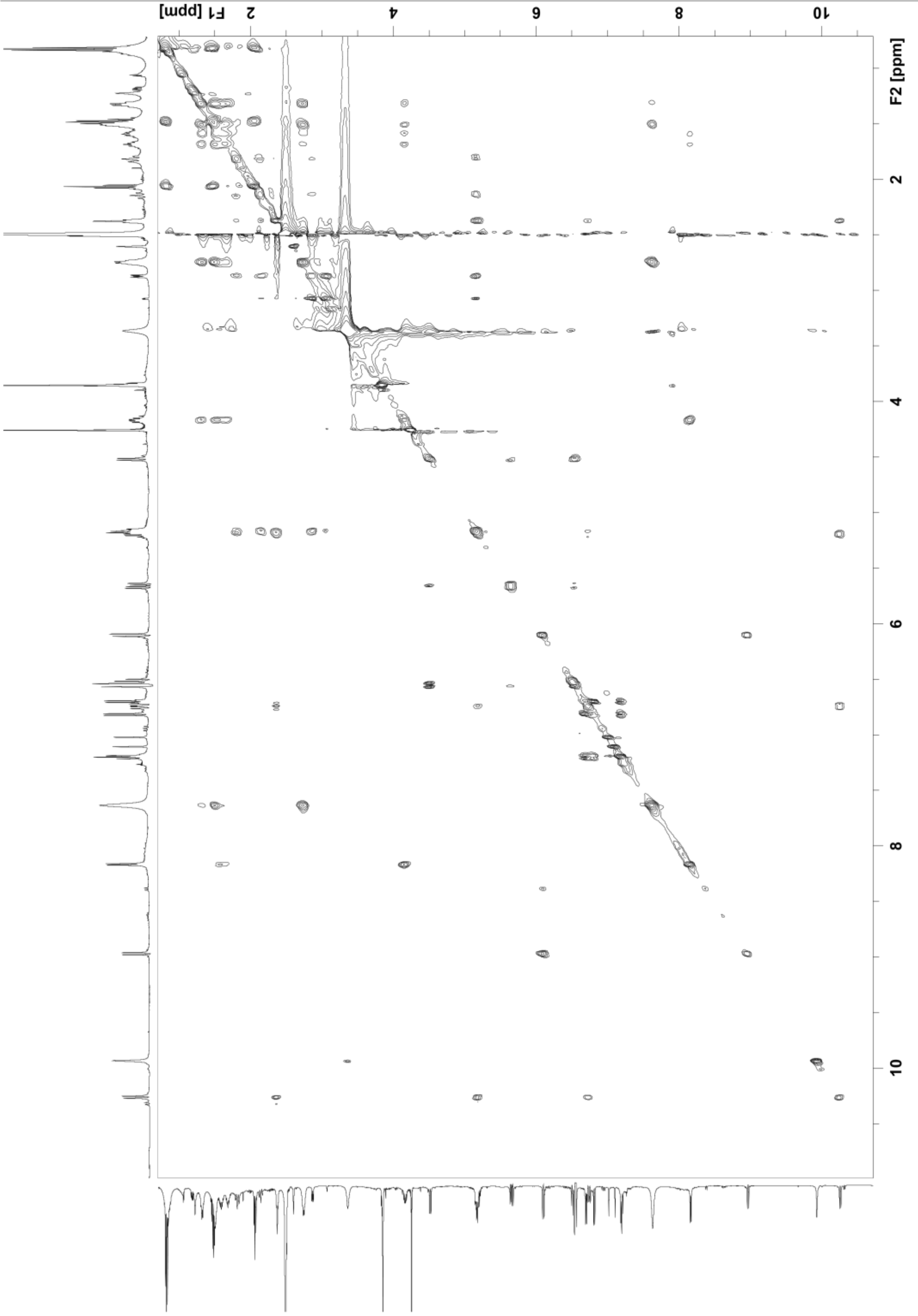

Figure S27. ^{1}H,^{1}H TOCSY NMR spectrum of necroxime B.

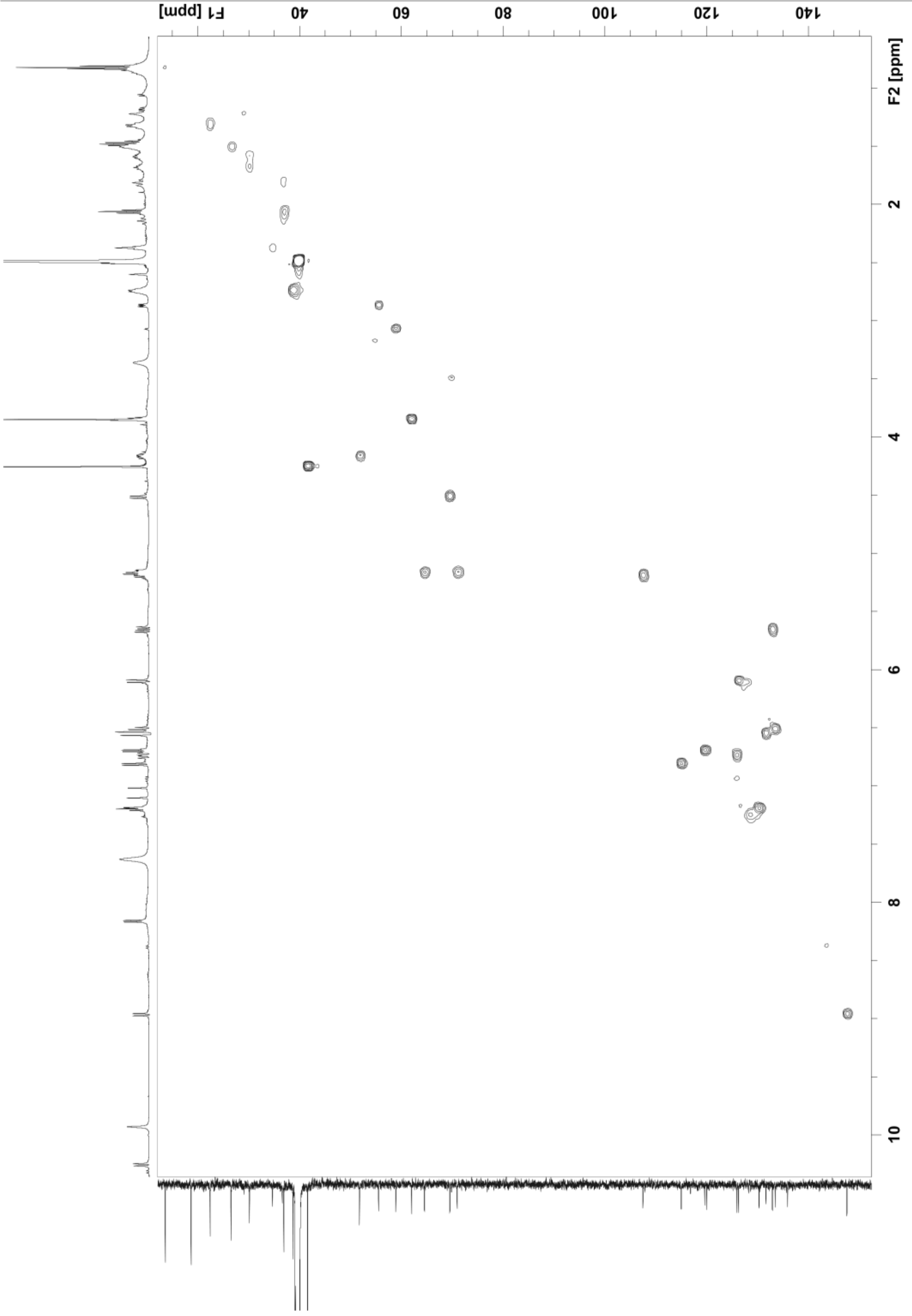

Figure S28. $^{1}H,^{13}C$ HSQC NMR spectrum of necroxime B.

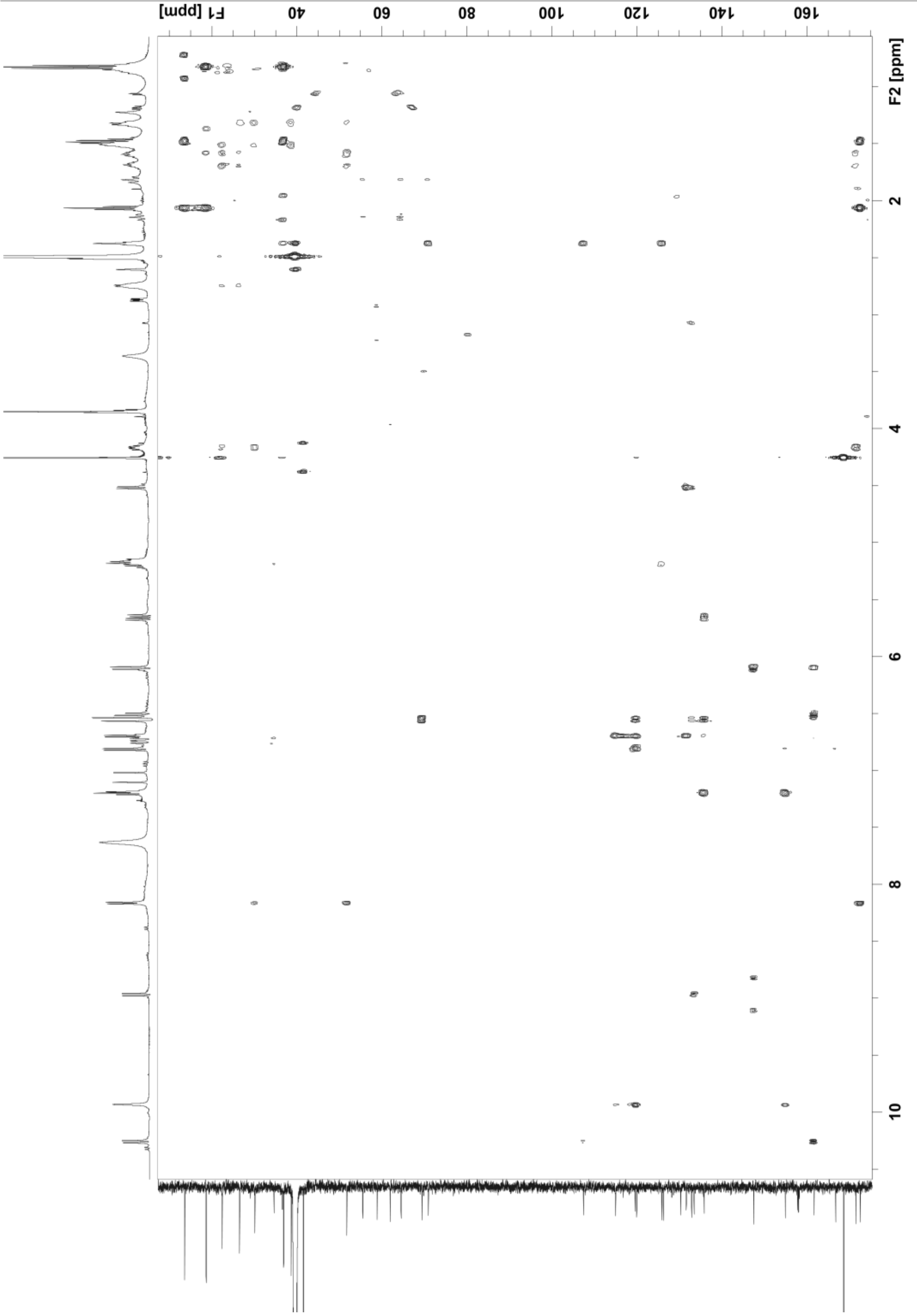

Figure S29. $^{1}H,^{13}C$ HMBC NMR spectrum of necroxime B.

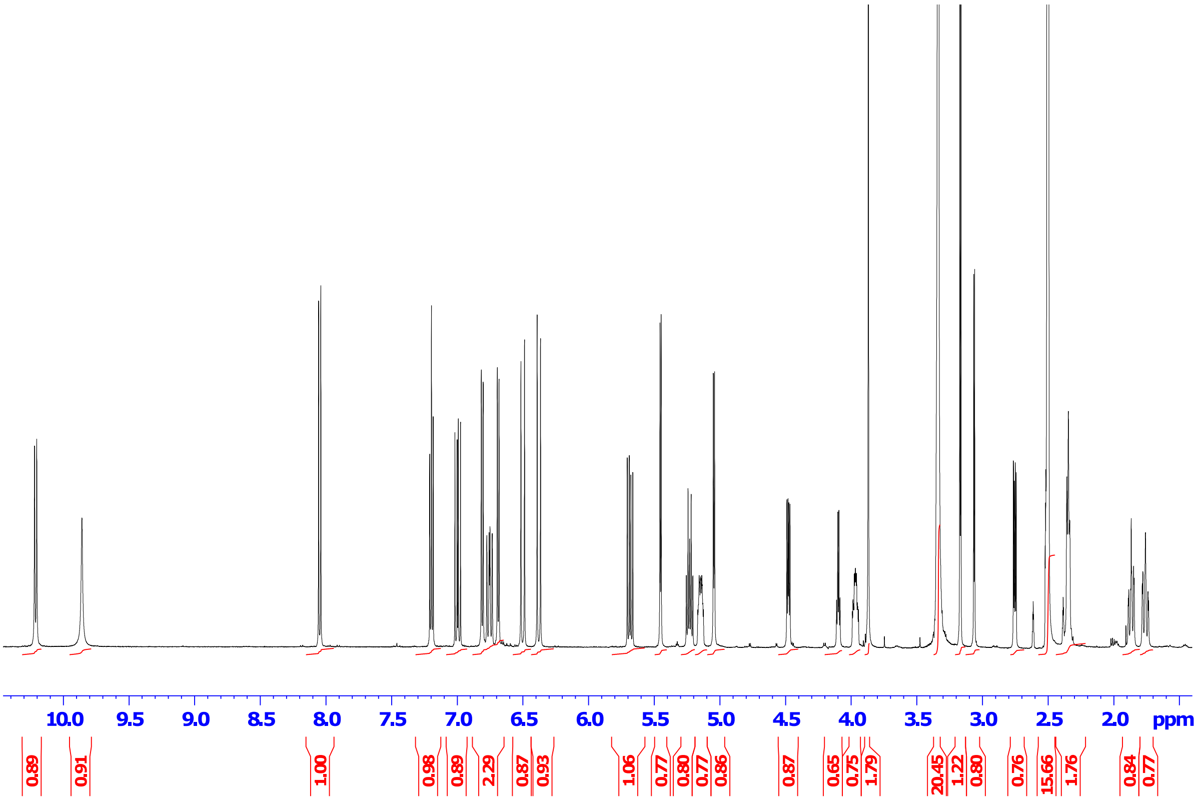

Figure S30. ^{1}H NMR spectrum of necroxime C.

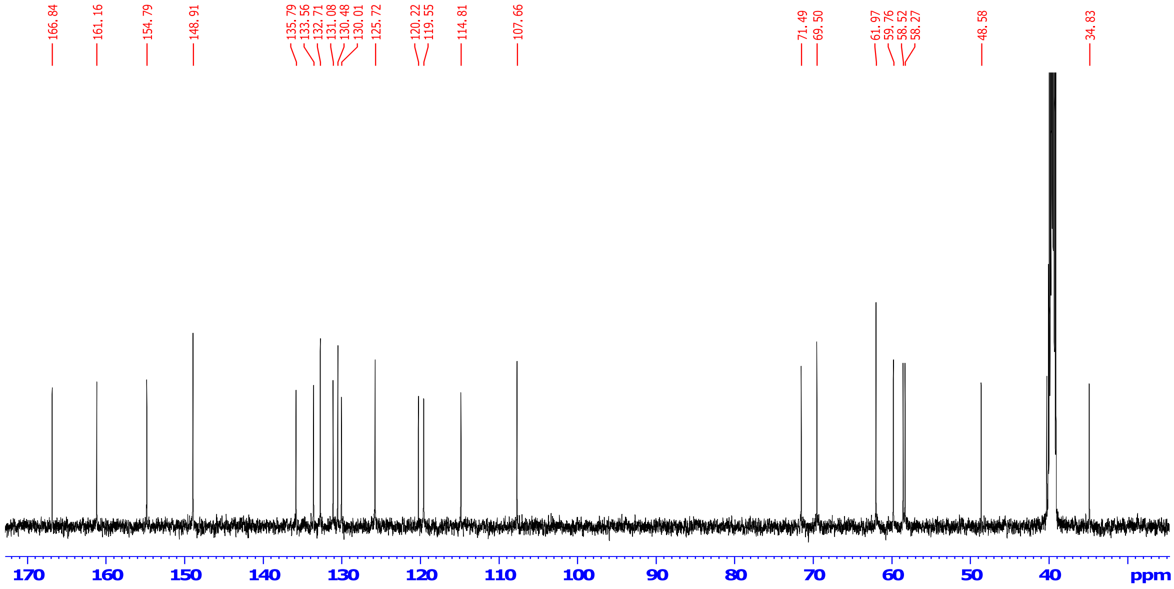

Figure S31. ^{13}C NMR spectrum of necroxime C.

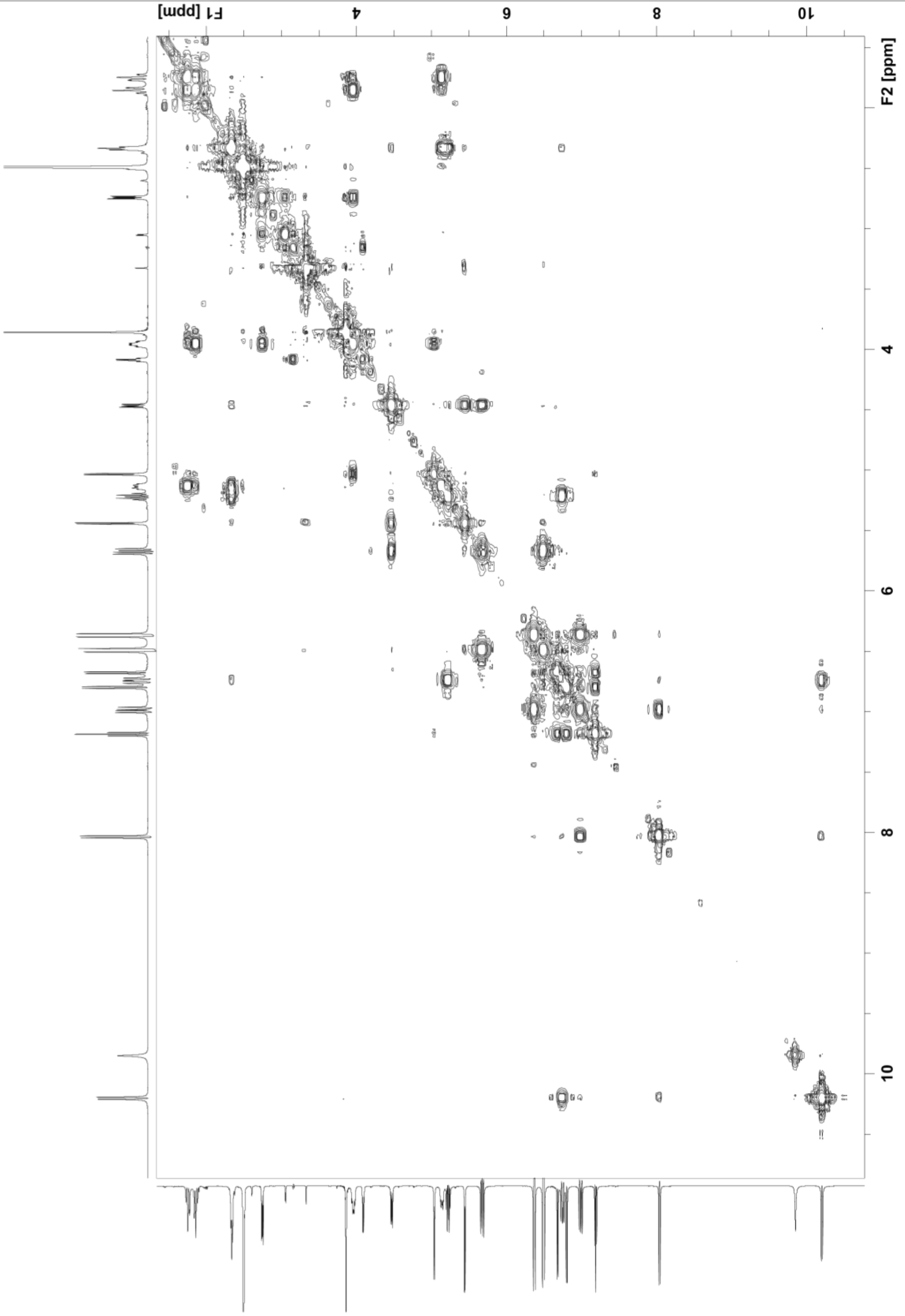

Figure S32. ^1H-^1H COSY NMR spectrum of necroxime C.

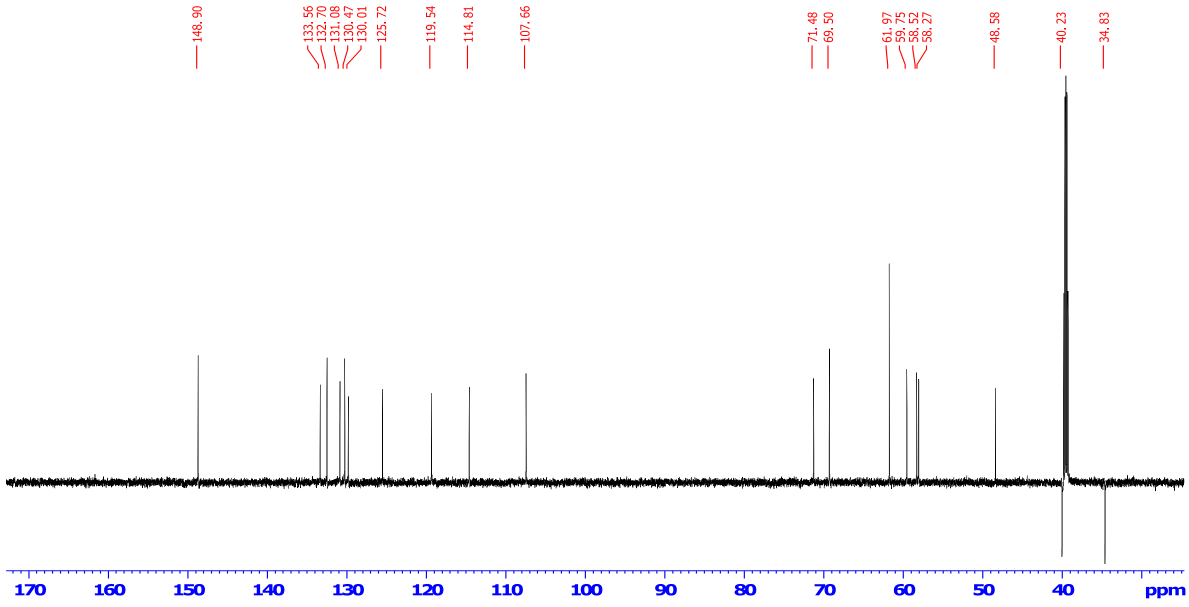

Figure S33. DEPT-135 NMR spectrum of necroxime C.

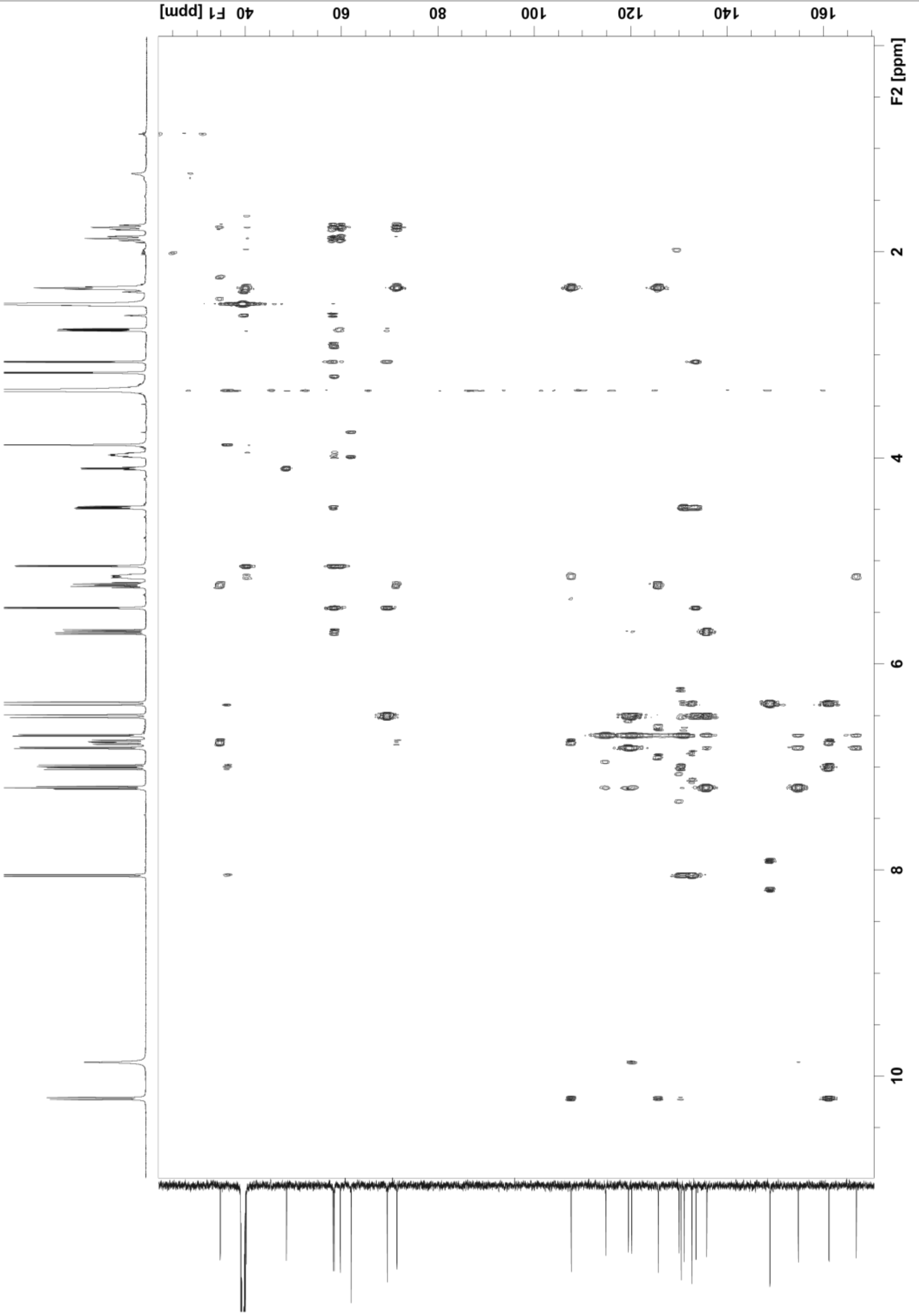

Figure S34. ^{1}H-^{13}C HMBC NMR spectrum of necroxime C.

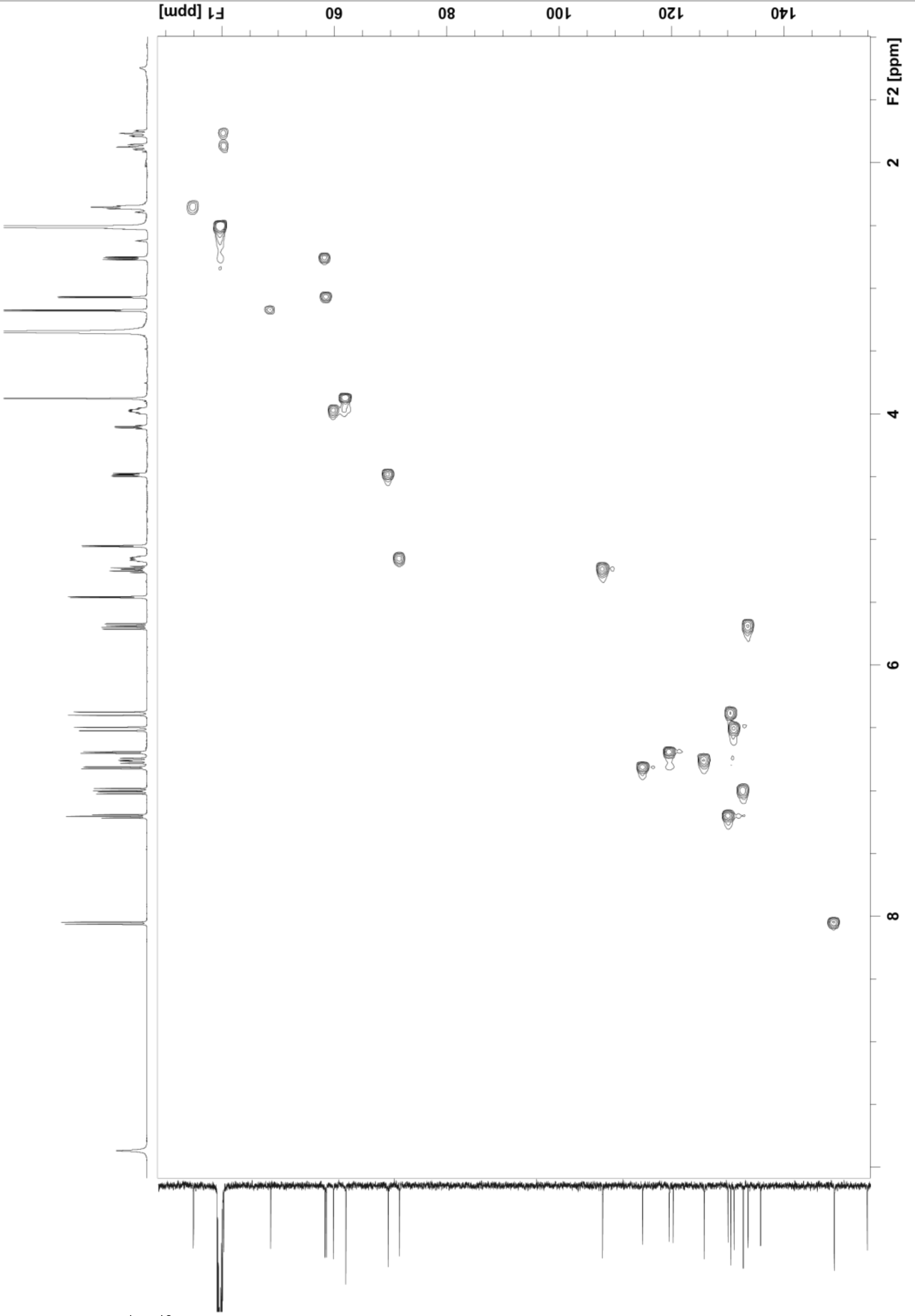

Figure S35. ^{1}H-^{13}C HSQC NMR spectrum of necroxime C.

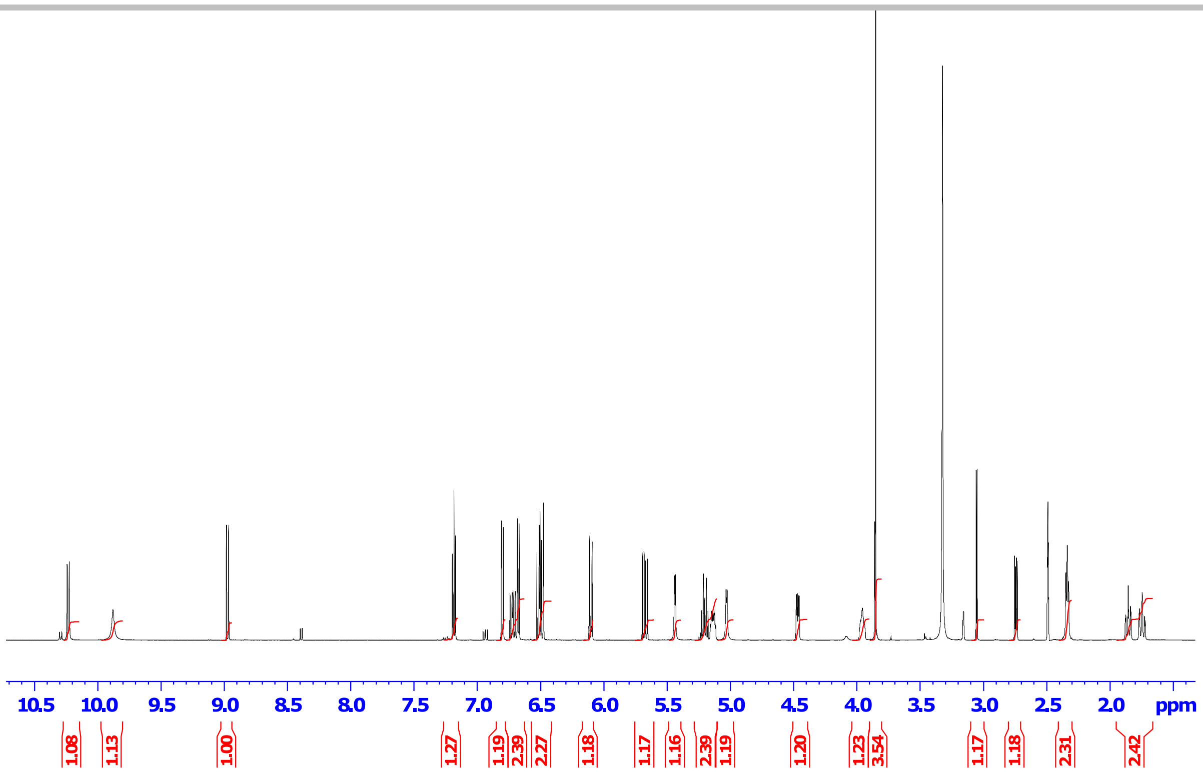

Figure S36. ^{1}H NMR spectrum of necroxime D.

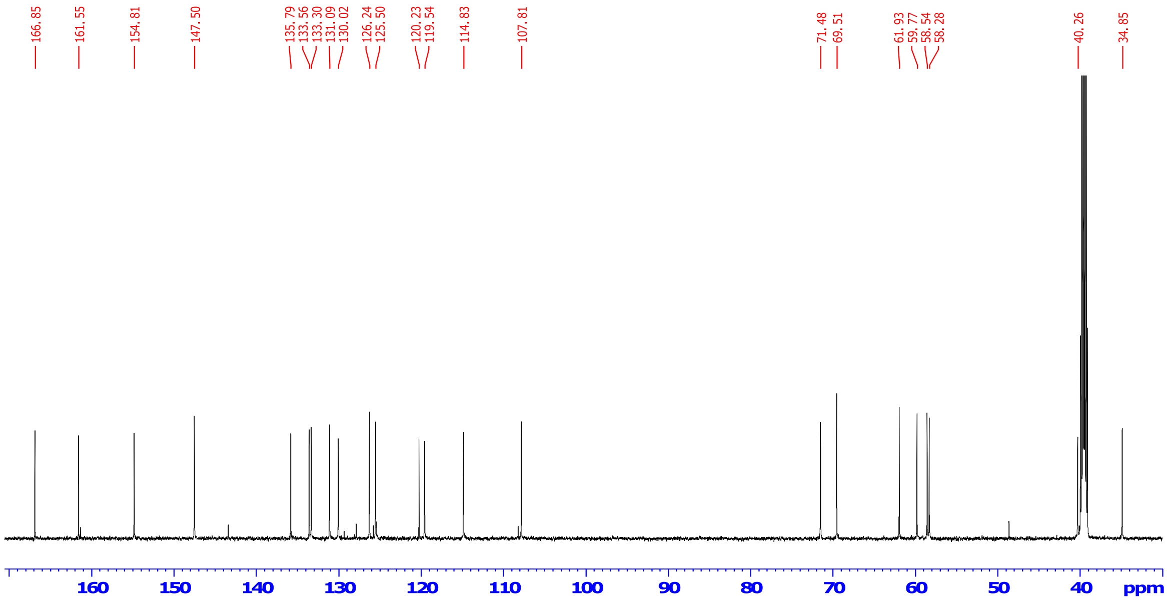

Figure S37. ^{13}C NMR spectrum of necroxime D.

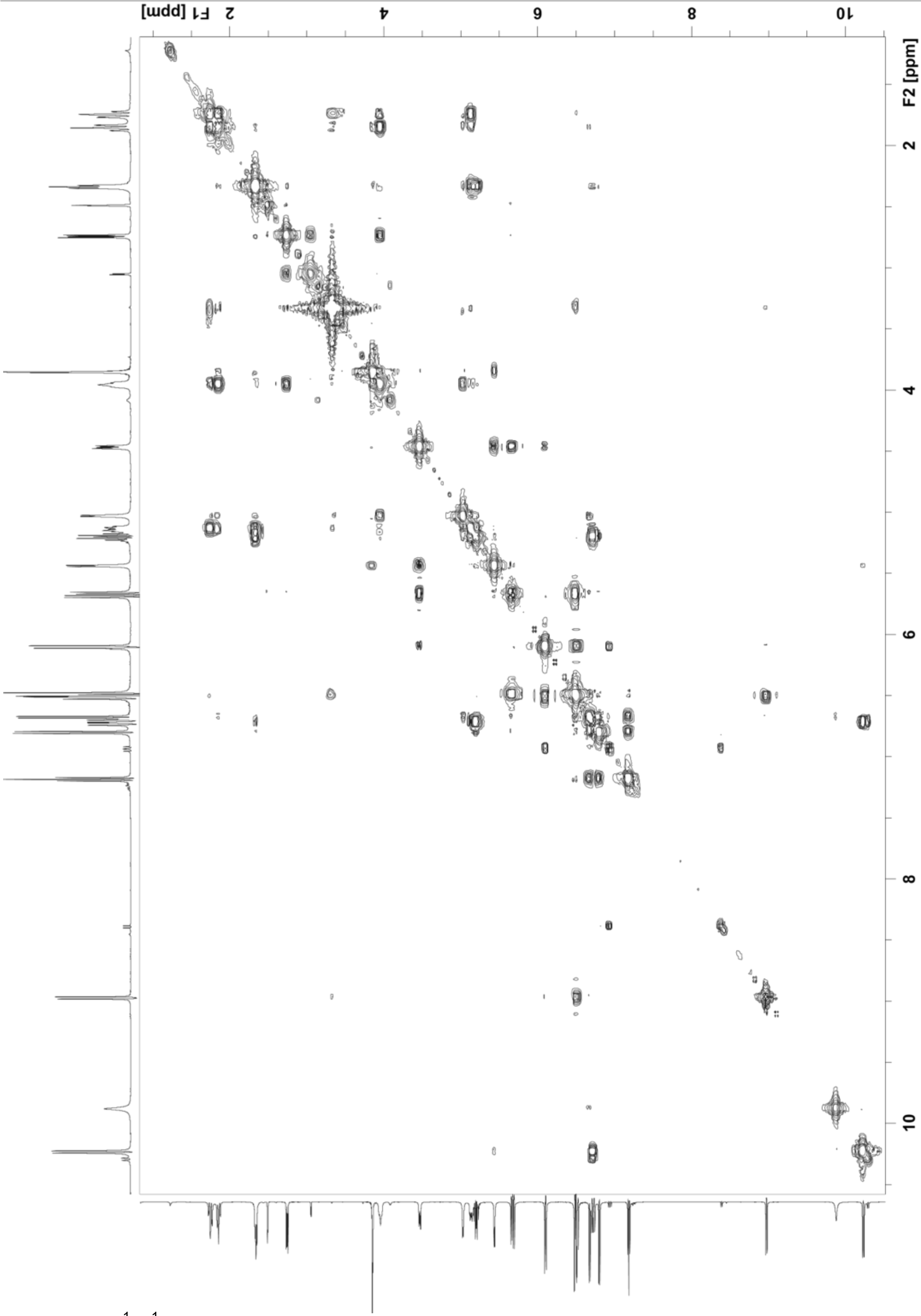

Figure S38. $^{1}H,^{1}H$ COSY NMR spectrum of necroxime D.

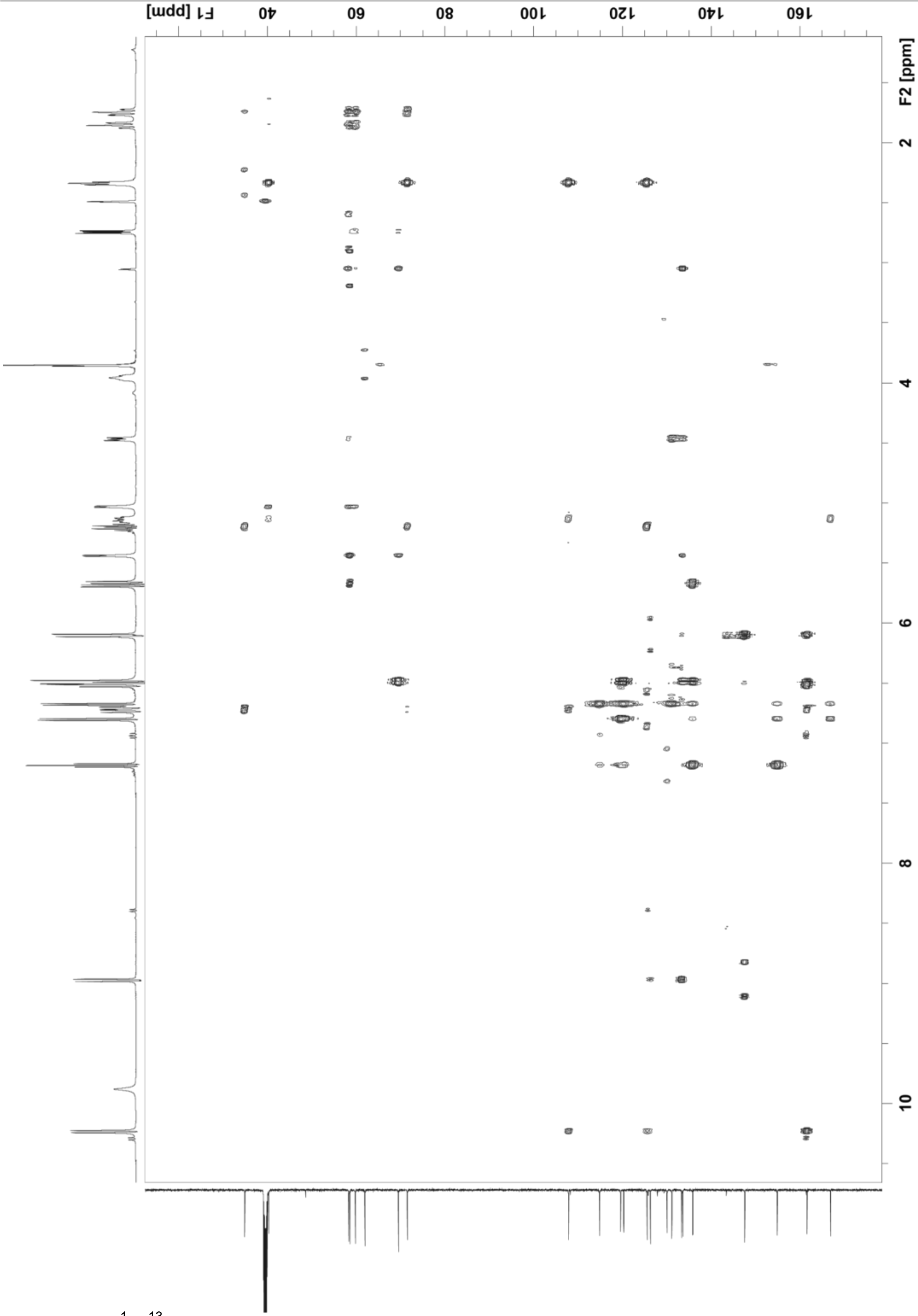

Figure S39. $^{1}H,^{13}C$ HMBC NMR spectrum of necroxime D.

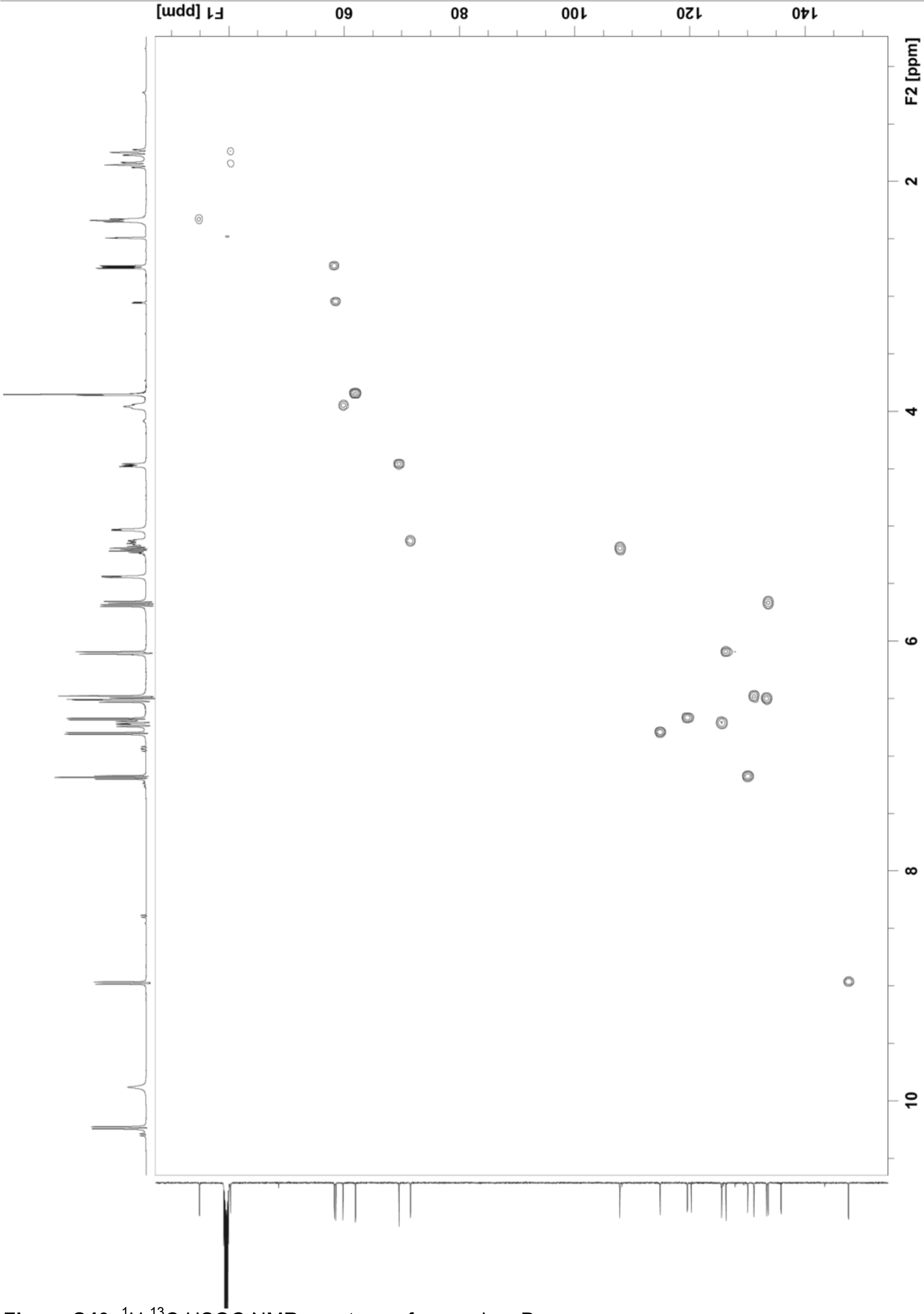

Figure S40. $^{1}H,^{13}C$ HSQC NMR spectrum of necroxime D.

References

[1] S. P. Niehs, B. Dose, K. Scherlach, M. Roth, C. Hertweck, *ChemBioChem* **2018**, *19*, 2167-2172.
[2] A. Bankevich, S. Nurk, D. Antipov, A. A. Gurevich, M. Dvorkin, A. S. Kulikov, V. M. Lesin, S. I. Nikolenko, S. Pham, A. D. Prjibelski, *J. Comput. Biol.* **2012**, *19*, 455-477.
[3] T. Seemann, *Bioinformatics* **2014**, *30*, 2068-2069.
[4] K. Blin, S. Shaw, K. Steinke, R. Villebro, N. Ziemert, S. Y. Lee, M. H. Medema, T. Weber, *Nucleic Acids Res.* **2019**.
[5] B. O. Bachmann, J. Ravel, *Methods Enzymol.* **2009**, *458*, 181-217.
[6] S. P. Niehs, B. Dose, K. Scherlach, S. J. Pidot, T. P. Stinear, C. Hertweck, *ACS Chem. Biol.* **2019**, *14*, 1811-1818.
[7] K. Ishida, T. Lincke, C. Hertweck, *Angew. Chem. Int. Ed.* **2012**, *51*, 5470-5474.
[8] B. Kusebauch, B. Busch, K. Scherlach, M. Roth, C. Hertweck, *Angew. Chem. Int. Ed.* **2010**, *49*, 1460-1464.
[9] F. Lohr, I. Jenniches, M. Frizler, M. J. Meehan, M. Sylvester, A. Schmitz, M. Gütschow, P. C. Dorrestein, G. M. König, T. F. Schäberle, *Chem. Sci.* **2013**, *4*, 4175-4180.
[10] D. C. Gay, P. J. Spear, A. T. Keatinge-Clay, *ACS Chem. Biol.* **2014**, *9*, 2374-2381.
[11] X.-H. Chen, J. Vater, J. Piel, P. Franke, R. Scholz, K. Schneider, A. Koumoutsi, G. Hitzeroth, N. Grammel, A. W. Strittmatter, *J. Bacteriol.* **2006**, *188*, 4024-4036.
[12] B. Schuster-Böckler, J. Schultz, S. Rahmann, *BMC Bioinform.* **2004**, *5*, 7.
[13] J. Trifinopoulos, L.-T. Nguyen, A. von Haeseler, B. Q. Minh, *Nucleic Acids Res.* **2016**, *44*, 232-235.
[14] G. E. Crooks, G. Hon, J.-M. Chandonia, S. E. Brenner, *Genome Res.* **2004**, *14*, 1188-1190.
[15] T. Nguyen, K. Ishida, H. Jenke-Kodama, E. Dittmann, C. Gurgui, T. Hochmuth, S. Taudien, M. Platzer, C. Hertweck, J. Piel, *Nat. Biotech.* **2008**, *26*, 225.
[16] K. Tamura, G. Stecher, D. Peterson, A. Filipski, S. Kumar, *Mol. Biol. Evol.* **2013**, *30*, 2725-2729.
[17] J. Trifinopoulos, L.-T. Nguyen, A. von Haeseler, B. Q. Minh, *Nucleic Acids Res.* **2016**, *44*, W232-W235.
[18] P. Caffrey, *ChemBioChem* **2003**, *4*, 654-657.
[19] a) J. He, M. Müller, C. Hertweck, *J. Am. Chem. Soc.* **2004**, *126*, 16742-16743; b) M. Ziehl, J. He, H. M. Dahse, C. Hertweck, *Angew. Chem. Int. Ed.* **2005**, *44*, 1202-1205.

Author contributions

S.P.N. purified compounds **3** and **4** and finalized structure elucidation including chemical derivatization and data evaluation. B.D. constructed knockout plasmids, performed genetic manipulation experiments and performed phylogenetic analyses. S.R. detected and purified compounds **1** and **2**. H.-M. Dahse performed antiproliferative and cytotoxicity assays. S.J.P. and T.S. conducted genomic sequencing and analyses of the genome. All authors contributed to writing of the manuscript. C.H. guided all aspects of the work and wrote the final draft.
